# *Xanthoceras sorbifolium* Bunge: A Review on Botany, Phytochemistry, Pharmacology, and Applications

**DOI:** 10.3389/fphar.2021.708549

**Published:** 2021-08-30

**Authors:** Erhuan Zang, Bin Qiu, Namuhan Chen, Caifeng Li, Qian Liu, Min Zhang, Yuchao Liu, Minhui Li

**Affiliations:** ^1^ Department of Pharmacy, Baotou Medical College, Baotou, China; ^2^ School of Chinese Materia Medica and Yunnan Key Laboratory of Southern Medicinal Resource, Yunnan University of Chinese Medicine, Kunming, China; ^3^ Pharmaceutical Laboratory, Inner Mongolia Hospital of Traditional Chinese Medicine, Hohhot, China; ^4^ Pharmaceutical Laboratory, Inner Mongolia Institute of Traditional Chinese Medicine, Hohhot, China; ^5^ Office of Academic Research, Qiqihar Medical University, Qiqihar, China; ^6^ Inner Mongolia Key Laboratory of Characteristic Geoherbs Resources Protection and Utilization, Baotou, China

**Keywords:** Xanthoceras sorbifolium, medicinal plants, phytochemistry, pharmacology, applications

## Abstract

*Xanthoceras sorbifolium* Bunge (Sapindaceae) is a native Chinese plant with promising applications as a biofuel feedstock and a source of novel drugs. Historical records and documents from different periods have mentioned the use of *X. sorbifolium* and its botanical constituents in treating diseases, highlighting its central role in Chinese and Mongolian traditional medicinal therapies. Phytochemical research has focused on the husks, leaves, trunks, and branches of this herb. A total of 278 chemical compounds have been isolated and divided into 8 categories: triterpenoids, flavonoids, phenylpropanoids, steroids, phenols, fatty acids, alkaloids, and quinones. Modern pharmacological studies on *X. sorbifolium* have demonstrated positive effects on learning and memory, as well as anti-inflammatory, anti-tumor, and anti-oxidative properties. This review provides a comprehensive analysis of the available research on *X. sorbifolium*, focusing on the relationship between chemical constituents, traditional uses, and pharmacological effects. We also assess the potential for therapeutic and other applications of this plant in support of further research and development of *X. sorbifolium*.

## Introduction

*Xanthoceras sorbifolium*, belonging to the family Sapindaceae and genus *Xanthoceras,* is a monotypic species widely distributed throughout China. The plant, commonly called the yellow horn or golden horn (Xu and Yu, 2010), is a valuable woody oil crop used to extract edible and medicinal ingredients, produce biofuels, and for greening of deserts. In China, *X. sorbifolium* was first recorded in the Chinese Materia Medica “*Jiu Huang Ben Cao*” (1406 AD) under the name “Wen Guan Hua.” It is used to treat arterial sclerosis, hyperlipidemia, hypertension, chronic hepatitis, and rheumatism (Wang, 1998; Li et al., 2007a). More importantly, each part of *X. sorbifolium* has a certain medicinal and health value and is used to prevent and treat diseases. The extract prepared from its husks has anti-inflammatory and anti-cancer properties; it also inhibits human immunodeficiency virus (HIV) protease and improves learning and memory, among other pharmacological effects (Zhang et al., 2016). The flower and calyx contain baicalin, which has antipyretic, sleep-inducing, anti-spasmodic, and anti-tumor effects. Their seeds can be used to prevent and cure arterial sclerosis (Wan et al., 2013). In Inner Mongolia, the trunks and branches were used to treat arthritis, as discussed in the Chinese Pharmacopeia in 1977 (Commission NP, 1977). Xanthoceraside is a triterpenoid saponin extracted from the husks of *X. sorbifolium*. It has many biological activities, such as improving learning and memory, and has anti-cancer and anti-inflammatory properties. Xanthoceraside may become a candidate for the prevention and treatment of Alzheimer’s disease (AD) (Yang C. Y. et al., 2016).

In addition to its medicinal value, *X. sorbifolium* has unique applications in the food and chemical industries, and in environmental protection (Wan et al., 2013). The seeds of *X. sorbifolium* are rich in unsaturated fatty acids and are used to prepare cooking oil. The kernels can be incorporated into seasoned dairy products or processed protein drinks. The leaves can also be used as tea (its protein content is higher than that of black tea), and the caffeine content is similar to that of flower tea (Wang, 1998). *Xanthoceras sorbifolium* can also be used in cosmetics and to make biodiesel. The husks of *X. sorbifolium* (which are considered by-products) can be used to produce chemical materials, such as activated carbon, furfural, xylitol, and alcohol (Yi et al., 2011). Furthermore, this herb is an excellent windbreak and a pioneer sand fixation species that is resistant to drought, wind, and sand. They are also easy to cultivate. Extensive *X. sorbifolium* plantations have been established in northern China to combat desertification. Other properties of this plant include cold tolerance, soil resistance, and high seed oil content. Therefore, it has become the preferred oil and eco-economic tree species for greening, returning farmland to forest, providing shelter against wind, and preventing sand erosion in mountainous areas (Bai et al., 2010). *Xanthoceras sorbifolium* has broad development prospects, especially in the fields of food, medicine, energy, and ecology. It is regarded as one of the most promising tree species for sustainable development in the 21st century.

In recent years, phytochemistry research has isolated 278 components from different sections of *X. sorbifolium*, including triterpenoids, flavonoids, phenylpropanoids, steroids, phenols, fatty acids, alkaloids, quinones, and others (Cheng et al., 2001; Wan et al., 2013; Yang C. Y. et al., 2016). These abundant bioactive components have a wide range of pharmacological activities (Yang L. et al., 2020; Zhang et al., 2020; Hao et al., 2021), including improved learning ability and memory (Ji et al., 2014; Rong et al., 2019), anti-inflammatory (Qi et al., 2013), anti-tumor (Wang et al., 2016a), antioxidant (Zhang et al., 2015; Yang CY. et al., 2016), anti-HIV (Li et al., 2007b), and vascular relaxation effects (Ma et al., 2000), as well as inhibition of pancreatic lipase activity (Geng et al., 2014). Increasing evidence regarding the medicinal value and excellent bioenergy value of *X. sorbifolium* highlights the need to evaluate its practical applications.

This review systematically summarizes the botanical and morphological characteristics, pharmacological effects, recorded medicinal history, and ethnic medicine applications of this herb. Through an extensive analysis of all relevant articles and books, we present the remarkable achievements and shortcomings of existing research, as well as some possible perspectives and trends for future studies on *X. sorbifolium*. This comprehensive review aims to provide a reference for future research, development, and utilization of *X. sorbifolium.*

## Methodology and Literature Search Strategy

The extensive literature search involved articles, papers, and books from different sources, such as Embase-Elsevier, PubMed, Science Direct, SciFinder Scholar, Google Scholar, Baidu Scholar, CNKI, and Web of Science. The search strategy was based on combining different keywords, such as *X. sorbifolium*, traditional uses, phytochemistry, pharmacology, and review. The literature search results included publications from 1960 to 2021 to ensure a systematic analysis of data on *X. sorbifolium.* Literature screening involved initially reading the keywords, title, and abstract of retrieved literature to identify the article’s relevance to this research. Potentially relevant literature was then downloaded, and the full text was assessed. Any relevant literature was included in the analysis. Any literature that did not conform to the theme was excluded. The chemical structural formula used in this manuscript was created using ChemDraw 18.0 (PerkinElmer, United States).

## Botany and Characteristics

**FIGURE 1:**
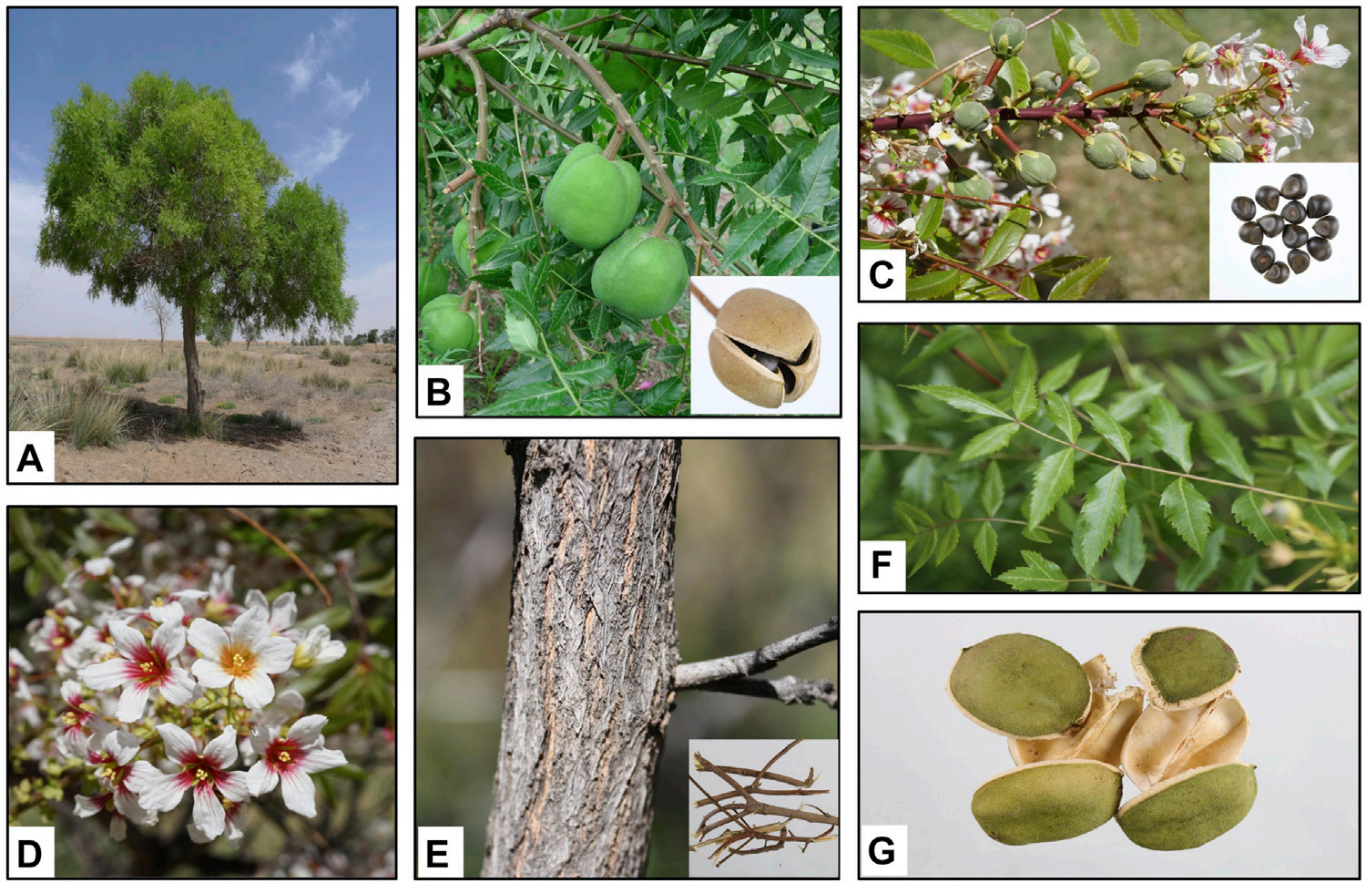
*Xanthoceras sorbifolium* Bunge. **(A)**. Whole plant; **(B)**. Fruits; **(C)**. Seeds; **(D)**. Flowers; **(E)**. Wood (The thunks and branches); **(F)**. Leaves; **(G)**. Husks.

*Xanthoceras sorbifolium* grows to 2–5 m in height with stout branchlets that are brownish red in color and glabrous with tile-liked bud scales arranged on the top and side buds. The leaf peduncle is 15–30 cm in length. There are 4–8 pairs of leaflets, membranous or papery, lanceolate or subovate. The lateral veins are slender and slightly raised on both sides. The inflorescence grows before or simultaneously with the leaves. The flowers are monoecious and the inflorescence is terminal. The axillary of the male inflorescences is 12–20 cm in length and erect with a short total pedicel and a residual bud scale at the base. The pedicel is 1.2–2 cm in length. The bracts are 0.5–1 cm long. The sepals are 6–7 mm long with gray hairs on both sides. The petals are white, but the base is purplish-red or yellow. The fruit consists of a capsule that is 5–6 cm in diameter with three seed compartments that are 1–1.5 cm in diameter. The number of seeds per compartment can vary from one to six. The seeds are black and shiny (Editorial Board of Flora of China, 1985; Xu and Yu, 2010). The different parts of *X. sorbifolium* are listed in Figure 1.

*Xanthoceras sorbifolium* grows in temperate and warm temperate zones, where the altitude ranges from 300 to 2000 m and the horizontal range is 28°34′–47°20′ E and 73°20′–120°25′ N. The species is mainly distributed in 18 provinces of China, including Inner Mongolia, Shaanxi, Shanxi, Hebei, and Henan. According to a resource survey, Chifeng in Inner Mongolia has the most concentrated populations and currently possesses the largest mangrove forest in China. The species is long-lived (up to 1,000 years), and it can grow in soil in pH ranges from 7 to 8.5. It can tolerate drought, low temperature, and soils that constitute clay, sand, or loam, including those that are alkaline, and of low fertility. It also grows well in deserted mountains, barren gullies, sandy lands, and steep hillsides (Mou et al., 2008; Xie et al., 2010).

## Phytochemistry

**FIGURE 2:**
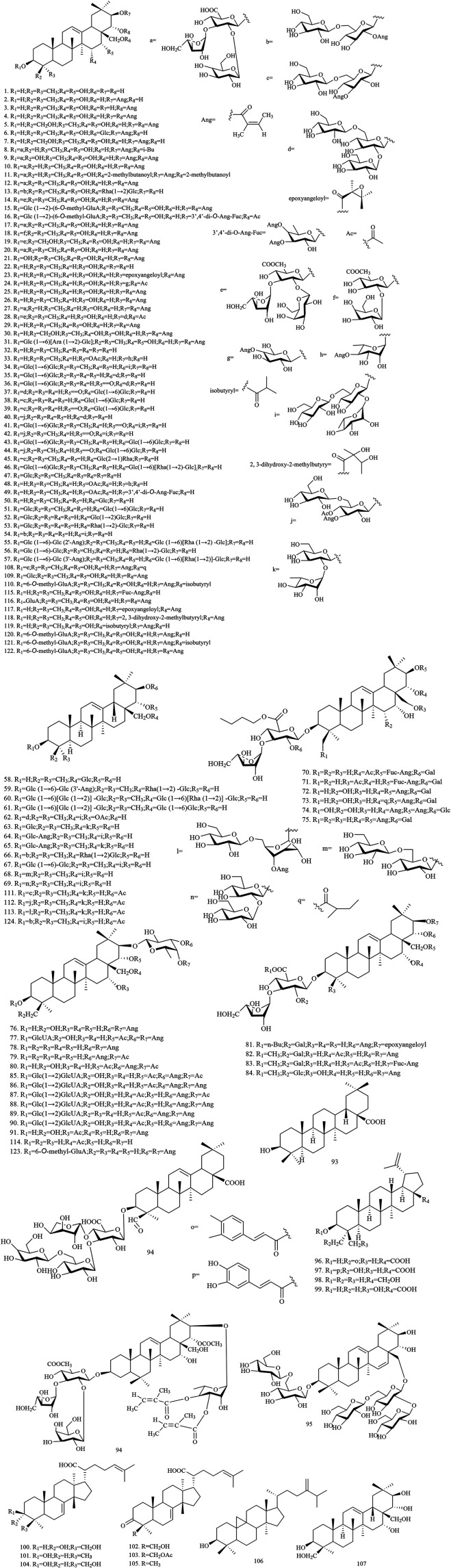
Structures of triterpenoids in *X. sorbifolium.*

**FIGURE 3:**
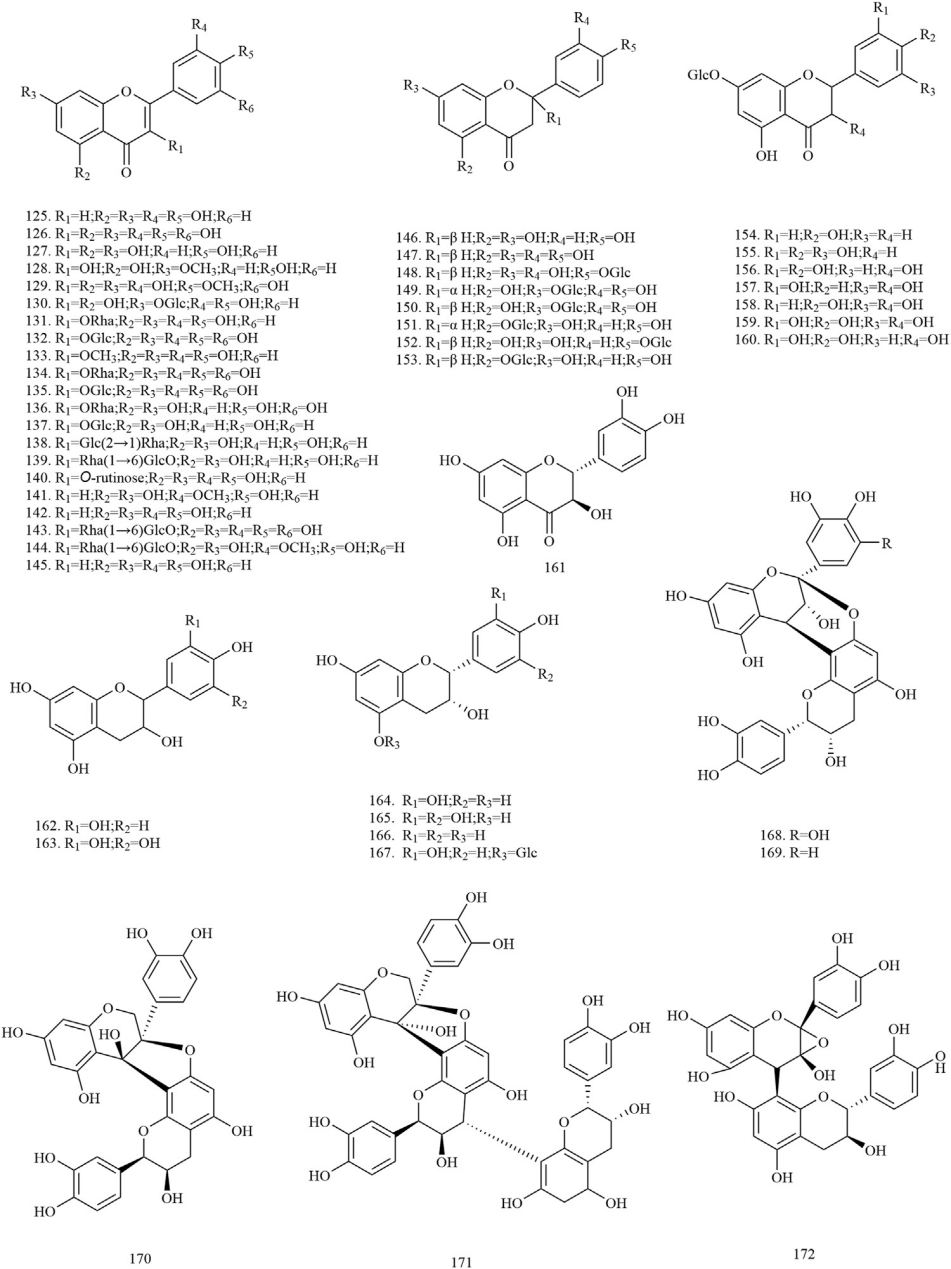
Structures of flavonoids in *X. sorbifolium*.

**FIGURE 4:**
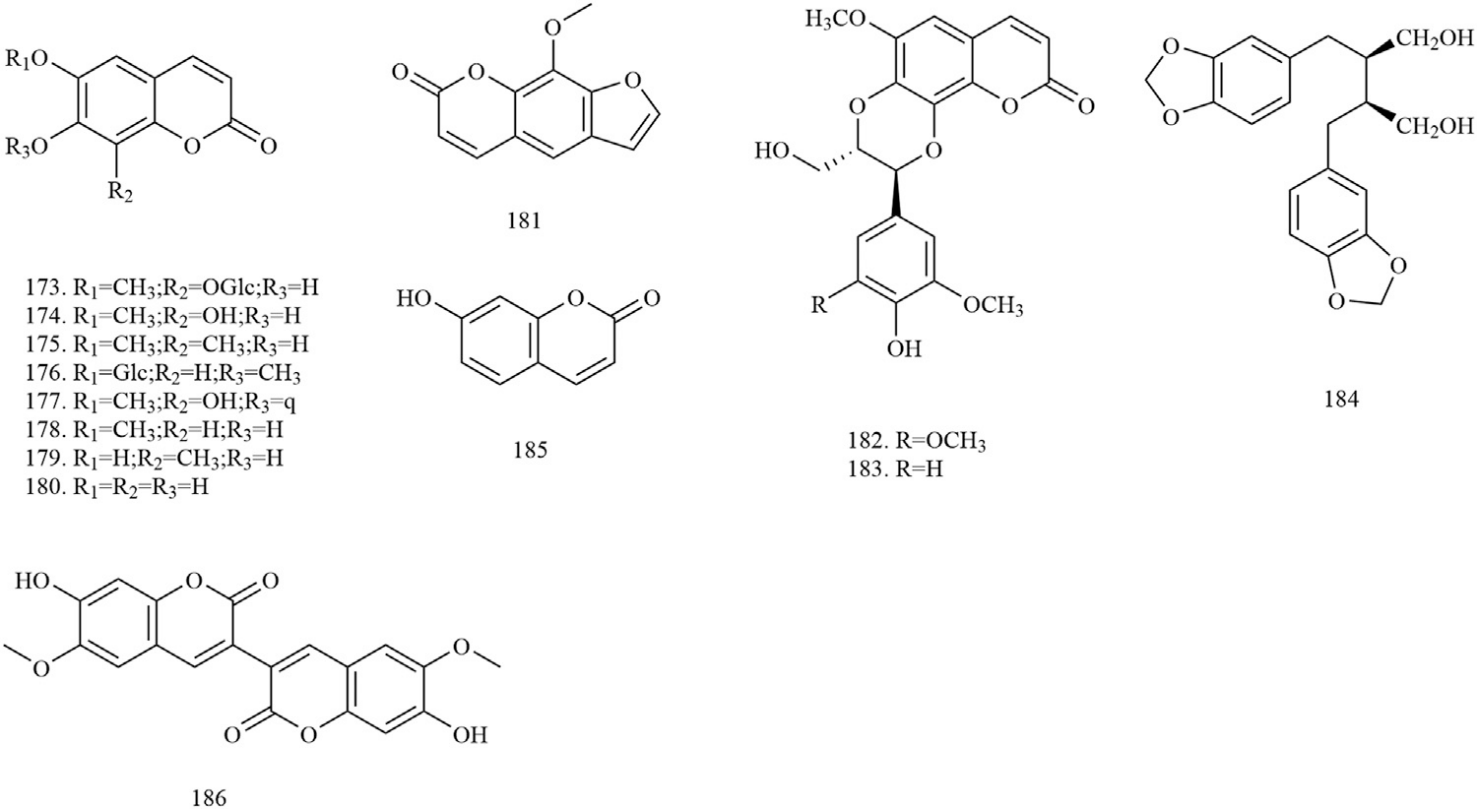
Structures of phenylpropanoids in *X. sorbifolium*.

**FIGURE 5:**
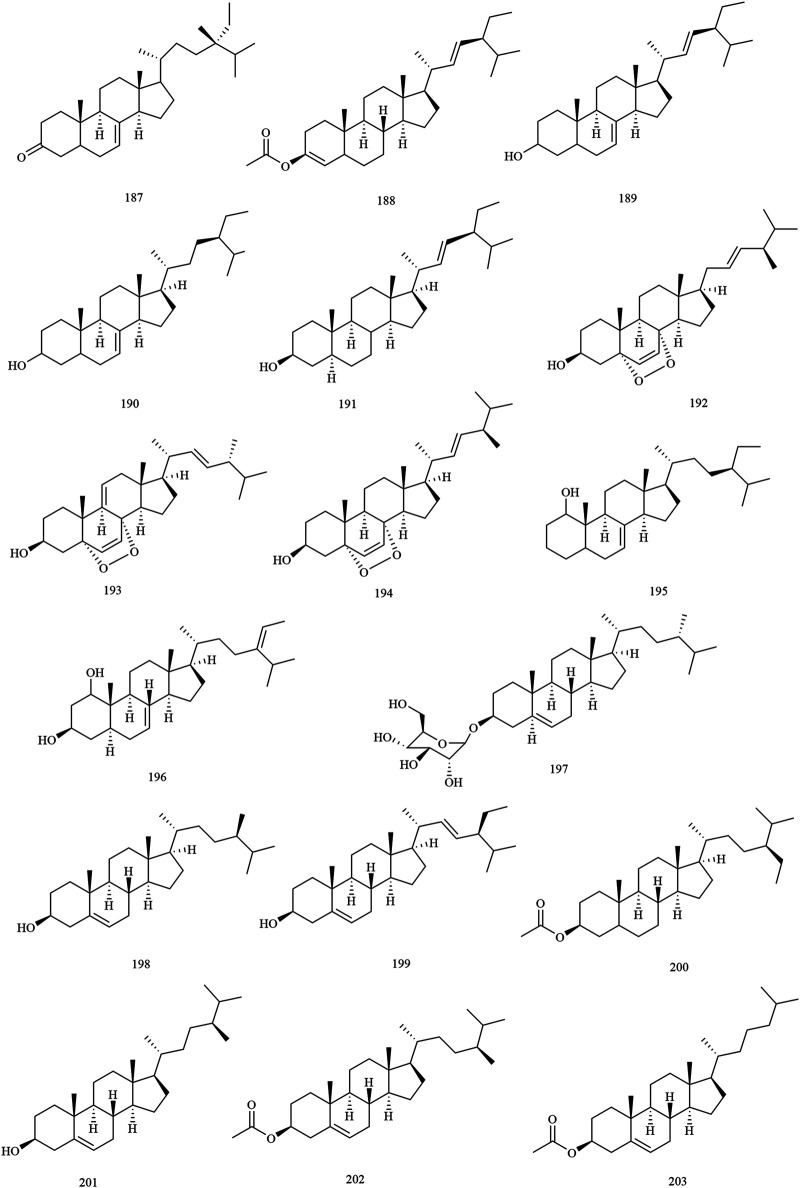
Structures of steroids in *X. sorbifolium*.

**FIGURE 6:**
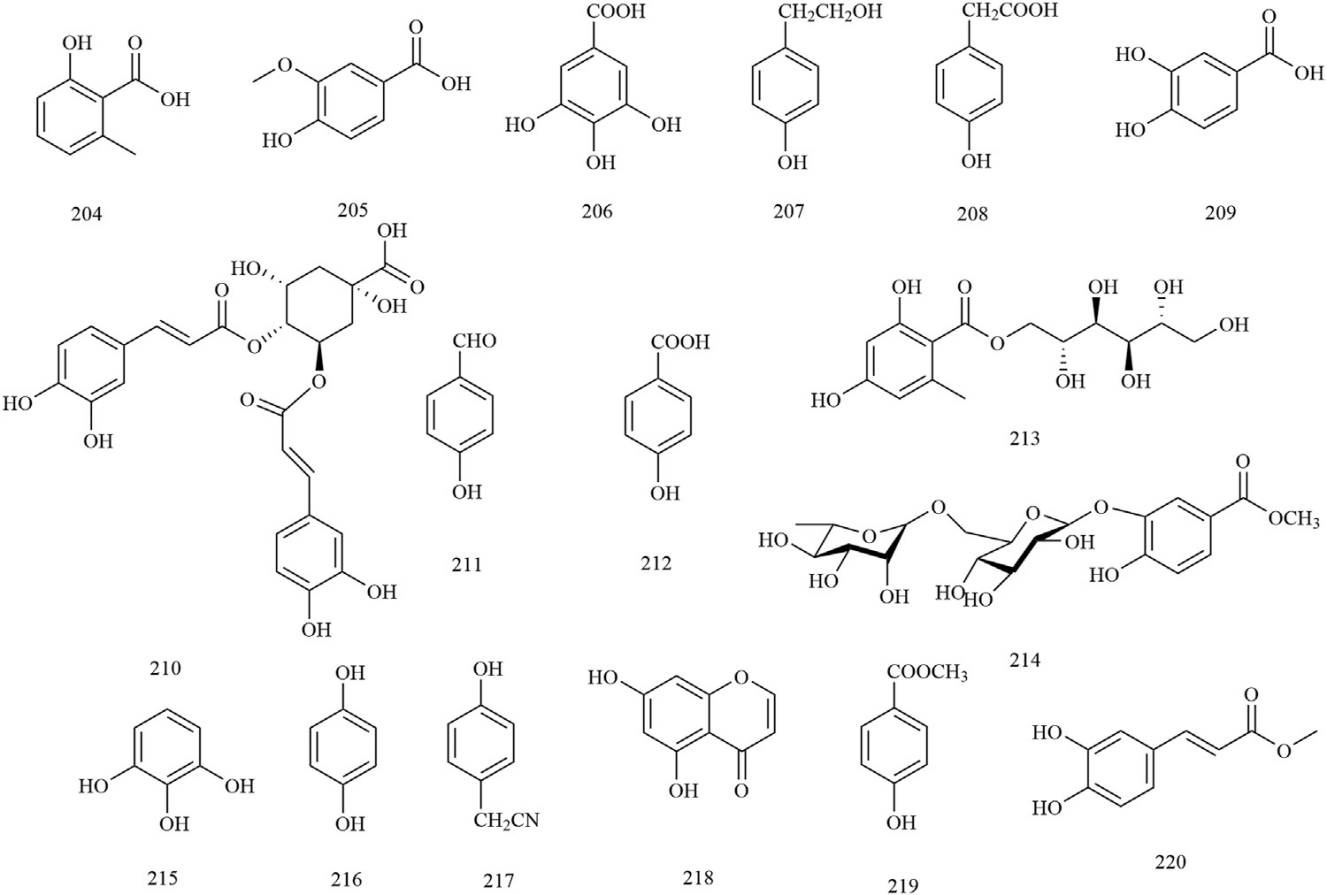
Structures of phenolic compounds in *X. sorbifolium*.

**FIGURE 7:**
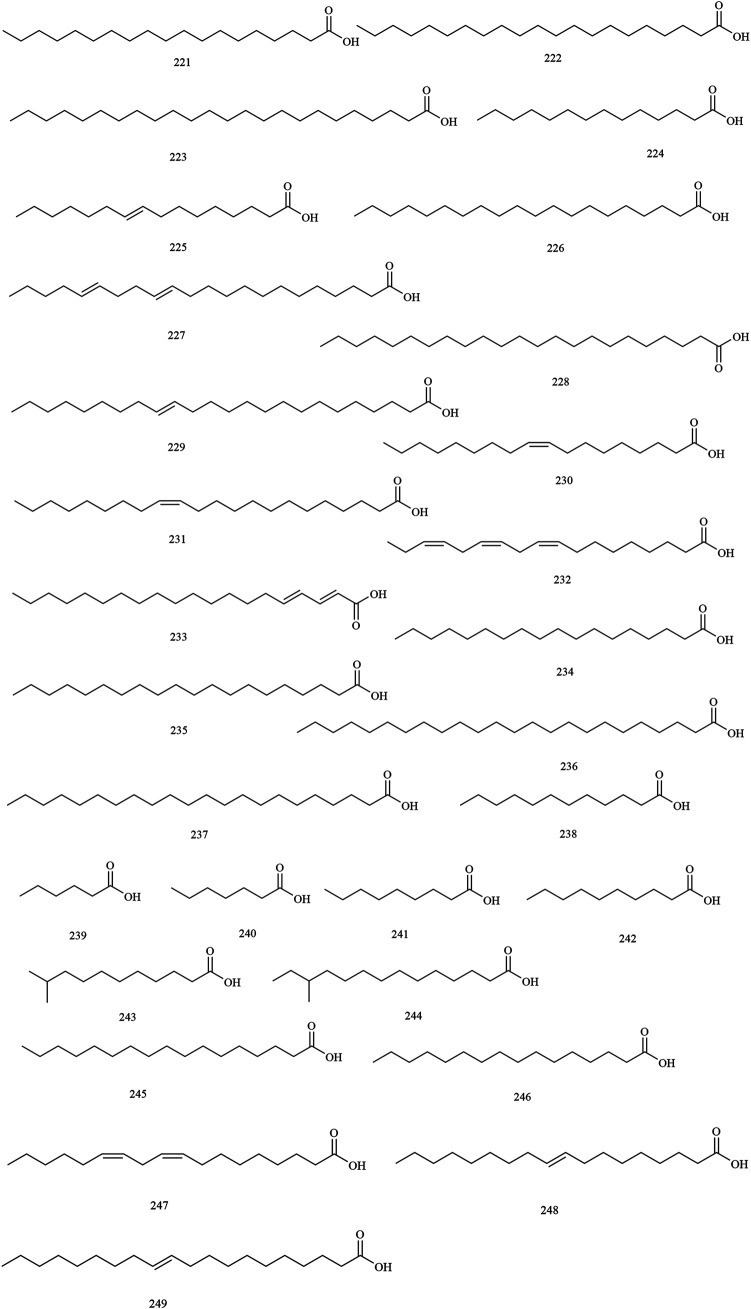
Structures of fatty acid compounds in *X. sorbifolium*.

**FIGURE 8:**
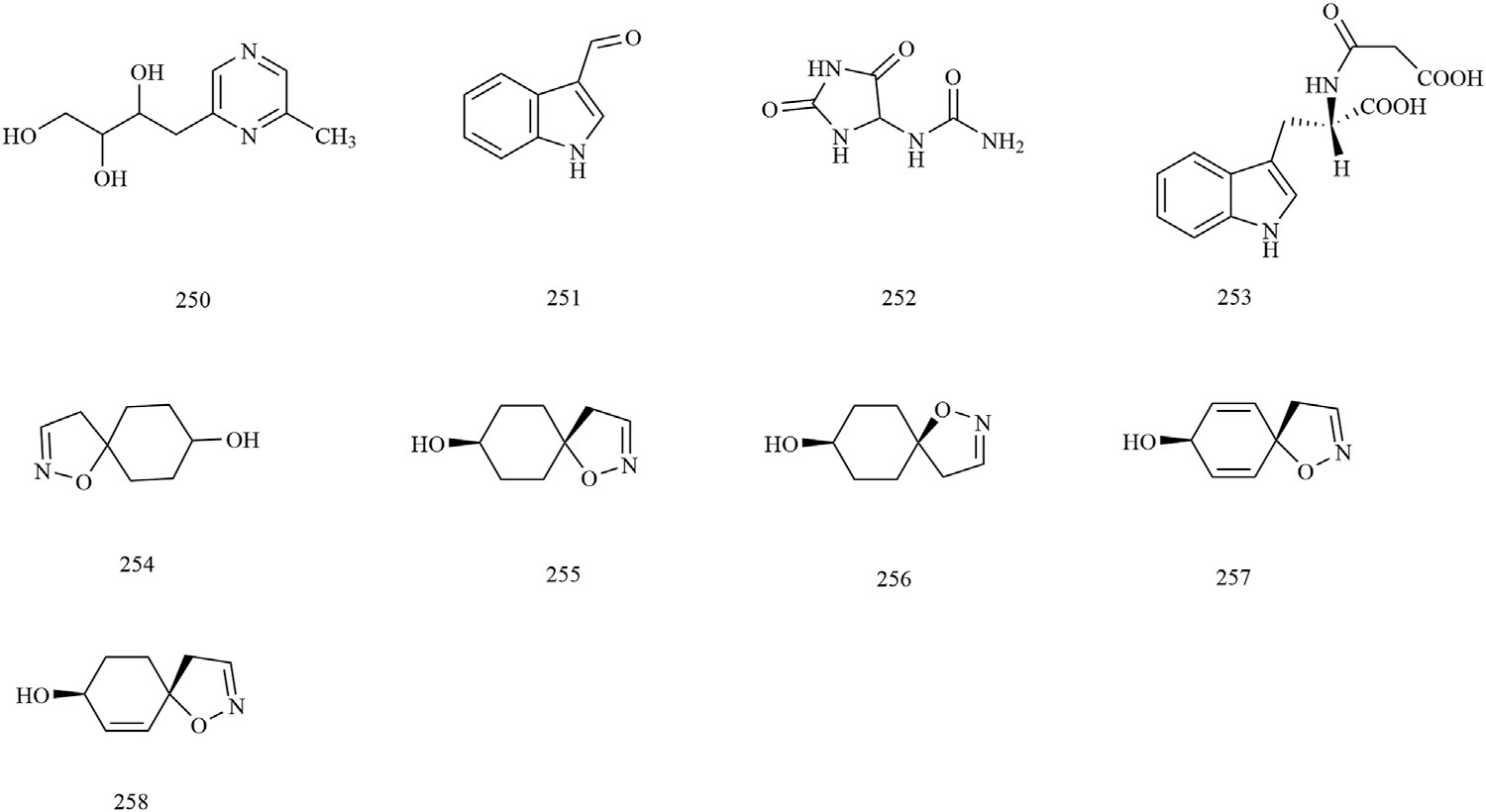
Structures of alkaloid compounds in *X. sorbifolium*.

**FIGURE 9:**
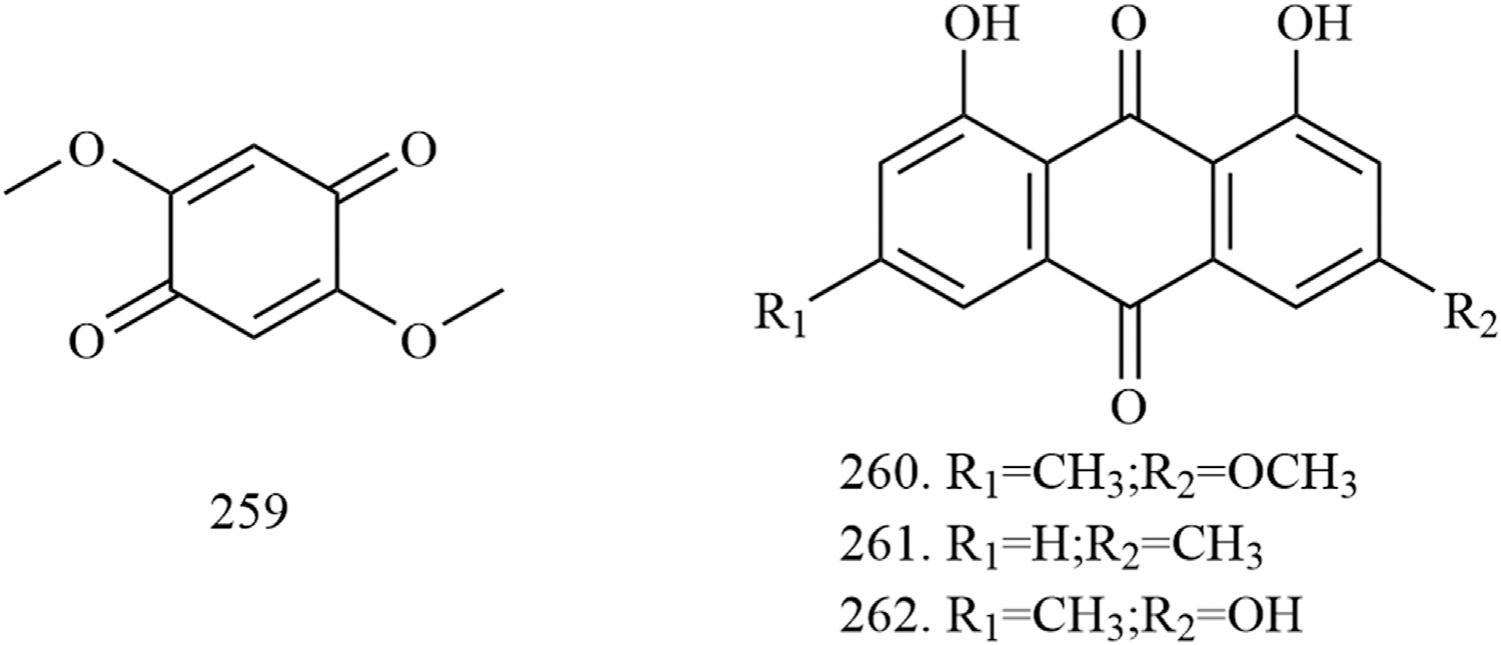
Structures of quinone compounds in *X. sorbifolium*.

**FIGURE 10:**
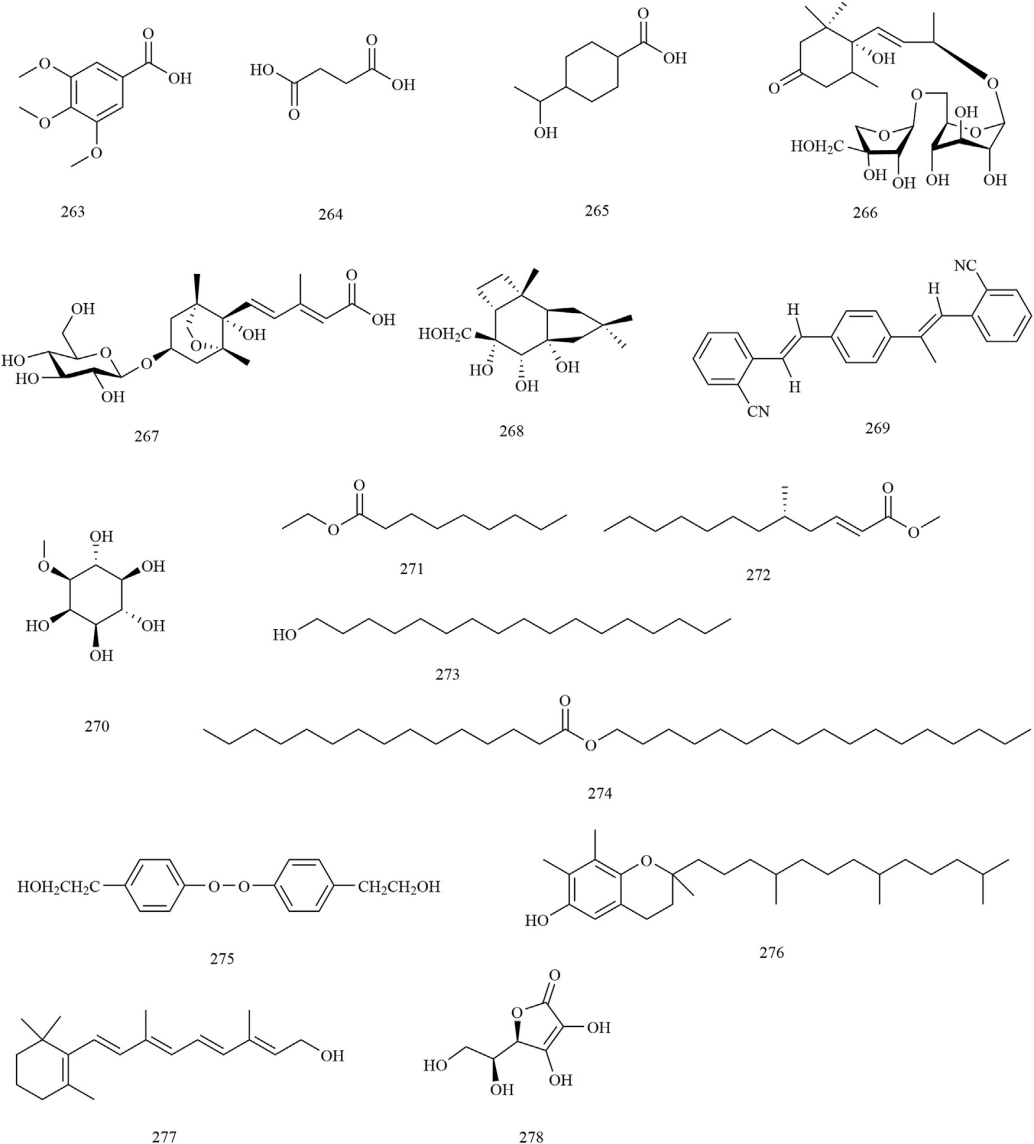
Structures of other compounds in *X. sorbifolium*.

Among the 278 compounds that have been isolated and identified from *X. sorbifolium*, triterpenes and flavonoids have been regarded as characteristic and main bioactive substances due to their variety, content, and pharmacological activities (Yang C. Y. et al., 2016). The structures and relevant references for these compounds are listed in Figures 2–10 and Table 1.

**TABLE 1:** The compounds isolated from *X. sorbifolium.*

NO.	Compounds classification and name	Plant parts	References
**Triterpenoids**			
01	R_1_-barrigenol	Husks/Carpophores	Li (2006a)
02	21-*O*-angeloyl-R_1_-barrigenol	Husks/Carpophores	Lin et al. (2004)
03	22-*O*-angeloyl-R_1_-barrigenol	Husks/Carpophores	Li (2006a)
04	21,22-di-*O*-angeloyl -R_1_-barrigenol	Husks/Carpophores	Li et al. (2005a)
05	21,22-di-*O*-angeloyl-24-hydroxy-R_1_-barrigenol	Husks/Carpophores	Li (2006a)
06	28-*O*-*β-*D-glucopyranosyl-21-*O*-angeloyl-R_1_-barrigenol	Husks/Carpophores	Wang et al. (2011); Li and Li (2014)
07	21-*O*-angeloyl-24-hydroxy-R_1_-barrigenol	Husks/Carpophores	Li and Li (2008)
08	xanifolia Y_0_	Husks	Chan et al. (2008)
09	xanifolia Y_2_	Husks	Chan et al. (2008)
10	xanifolia Y_3_	Husks	Chan et al. (2008)
11	xanifolia Y_7_	Husks	Chan et al. (2008)
12	3-*O*-(3-*O*-*α*-L-arabinofuranosyl-2-*O*-*β*-D-galactopyranosyl)-(6-*O*-methyl)-*β*-D-glucuronopyranosyl-21,22-di-*O*-angeloyl-R_1_-barrigenol	Husks/Carpophores	Wang et al. (2011)
13	3-*O*-*β*-D-galactopyranosyl-(1→6)-(2-*O*-angeloyl)-*β*-D-glucopyranosyl saniculagenic C-28-*O*-*α*-L-rhamnopytanosyl-(1→2)-*β*-D-glucopyranoside	Husks	Wan et al. (2013)
14	3-*O*-[*β*-D-galactopyranosyl (1→2)]-*α*-L-arabinofuranosyl (1→3)-*β*-D-methyl glucuronic acid-21, 22-*O*-diangeloyl-3*β*, 15*α*, 16*α*, 21*β*, 22*α*, 28*β*-hexahydroxyl-olean-12-ene	Husks	Guo et al. (2009)
15	3-*O*-(2-*O*-*β*-D-glucopyranosyl)-(6-*O*-methyl)-*β*-D-glucuronopyranosyl-21,22-di-*O*-angeloy-R_1_-barrigenol	Leaves	Xiao et al. (2013)
16	3-*O*-(2-*O*-*β*-D-glucopyranosyl)-(6-*O*-methyl)-*β*-D-glucuronopyranosyl-21-*O*-(3′,4′-di-*O*-angeloyl)-*β*-D-fucopyranosyl-22-*O*-acetyl-R_1_-barrigenol	Leaves	Xiao et al. (2013)
17	xanthoceraside	Trunks and branches	Liu et al. (2013)
18	6′-methylether-*O*-xanifolia-Y_5_	Husks	Wang et al. (2016a)
19	6′-methylester-*O*-xanifolia-Y_2_	Husks	Wang et al. (2016a)
20	xanifolia Y	Husks	Wang et al. (2016a)
21	xanifolia ACH-Y	Husks	Wang et al. (2016a)
22	barringtogenol C	Husks	Li (2006a)
23	22-*O*-angeloyl-21-*O*-epoxyangeloyl-barringtogenol C	Husks	Li (2006a)
24	22-*O*-acetyl-21-*O*-(4′-*O*-angeloyl)-*β*-D-fucopyranosyl theasapogenol B	Carpophores	Li et al. (2016a); Li et al. (2006c)
25	21,22-di-*O*-angeloyl-barringtogenol C	Husks	Li et al. (2005a)
26	xanifolia Y_8_	Husks	Chan et al. (2008)
27	xanifolia Y_10_	Husks	Chan et al. (2008)
28	*3- O*-[*β*-D-galactopyranosyl (1→2)]-*α-*L-arabinofuranosyl (1→3)-*β*-D-methyl glucuronic acid-21-*O*-(3,4-diangeloyl)-*α*-L-rhamnose-3*β*,16*α*,21*β*,22*α*,28*β*-pentahydroxyl-22-acetoxy-olean-12-ene	Husks	Guo et al. (2009)
29	21,22-diangeloyl-R_1_-barrigenol	Husks	Li et al. (2005b)
30	21,22-diangeloyl-24-hydroxy-R_1_-barrigenol	Husks	Li et al. (2005b)
31	3-*O*-*β*-D-glucopyranosyl (1→6)[*α*-L-*α*-rabinofuranosy (1→2)]-*β*-D-glucopyranosyl-21,22-di-*O*-angeloyl-R_1_-barringenol	Leaves	Xiao et al. (2013)
32	16-deoxybarringtogenol C	Husks/Carpophores	Li (2006a)
33	*16- O*-acetyl-21-*O*-(4-*O*-angeloyl-*α*-*L*-rhamnopytanosyl)-barringtogenol C	Husks/Carpophores	Li (2006a)
34	3-*O*-*β*-D-glucopyranosyl (1→6)-*β*-D-glucopyranosyl- 28-*O*-*β*-D-glucopyranosyl (1→6)[*α*-L-rhamnopyranosyl (1→2)]-*β*-D-glucopyranosyl-16-deoxybarringtogenol C	Leaves	Xiao et al. (2013)
35	sorbifoliaside A	Husks/Carpophores	Yu et al. (2012a)
36	sorbifoliaside B	Husks/Carpophores	Yu et al. (2012a)
37	sorbifoliaside C	Husks/Carpophores	Yu et al. (2012a)
38	sorbifoliaside D	Husks/Carpophores	Yu et al. (2012a)
39	sorbifoliaside E	Husks/Carpophores	Yu et al. (2012a)
40	sorbifoliaside F	Husks/Carpophores	Yu et al. (2012a)
41	sorbifoliaside G	Seed oil residue	Yu et al. (2012b)
42	sorbifoliaside H	Seed oil residue	Yu et al. (2012b)
43	sorbifoliaside I	Seed oil residue	Yu et al. (2012b)
44	sorbifoliaside J	Seed oil residue	Yu et al. (2012b)
45	3-*O*-[*β*-D-glucopyranosyl (1→6)](3′-*O*-angeloyl)-*β*-*D*-glucopyranosyl-28-*O*-[*α*-L-rhamnopytanosyl (1→2)]-*β*-*D*-glucopyranosyl-16-deoxybarringtogenol C	Carpophores	Li et al. (2008)
46	*3- O*-*β*-D-glucopyranosyl (1→6)-*β*-D-glucopyranosyl-28-*O*-*β*-D-glucopyranosyl (1→6)[*α*-L-rhamnopytanosyl (1→2)]-*β*-D-glucopyranosyl-16-deoxybarringtogenol C	Husks/Carpophores	Li et al. (2016a)
47	3-*O*-*β*-*D*-glucopyranosyl-16-deoxybarringtogenol C	Husks/Carpophores	Wang et al. (2011); Li and Li (2014)
48	16-*O*-acetyl-21-*O*-(4-*O*-angeloyl-*α*-L-rhamnopyranosyl)-barringtogenol C	Husks	Li (2006a)
49	16-*O*-acetyl-21-*O*-(3′,4′-di-*O*-angeloyl)-*β*-*D-*fucopyranosyl theasapogenol B	Husks/Carpophores	Li et al. (2007d)
50	28-*O*-*β*-*D*-glucopyranosyl-16-deoxybarringtogenol C	Husks	Li (2006a); Li et al. (2007c)
51	3-*O*-*β*-*D*-glucopyranosyl,28-*O*-[*α*-*L*-rhamnosyl (1→2)]-*β*-*D*-glucopyranosyl-16-deoxybarringtogenol C	Carpophores	Li et al. (2008)
52	3-*O*-*β*-D-glucopyranosyl-28-*O*-[*β*-D-glucopyranosyl (1→2)]-*β*-D-glucopyranosyl-21*β*,22*α*-dihydroxyl-olean-12-ene	Husks	Cui et al. (2012)
53	3-*O*-*β*-D-glucopyranosyl-28-*O*-[*α*-L-rhamnopyranosyl (1→2)]-*β*-D-glucopyranosyl-21*β*,22*α*-dihydroxyl-olean-12-ene	Husks	Cui et al. (2012)
54	3-*O*-*β*-*D*-glucopyranosyl (1→6)-[angeloyl (1→2)]-*β*-*D*-glucopyranosyl-28-*O*-*α*-*L*-rhamnopyranosyl (1→2)-[*β*-*D*-glucopyranosyl (1→6)]-*β*-*D*-glucopyranosyl-21*β*,22*α*-dihydroxylolean-12-ene	Husks/Carpophores	Cui et al. (2012)
55	*3- O*-*β*-*D*-glucopyranosyl (1→6)-(2′-angeloyl)-*β*-*D*-glucopyranosyl-28-*O*-*β*-*D*-glucopyranosyl (1→6)[*α*-*L*-rhamnopytanosyl (1→2)]-*β*-*D*-glucopyranosyl-16-deoxybarringtogenol C	Leaves	Xiao et al. (2013)
56	3-*O*-*β*-D-glucopyranosyl (1→6)-*β*-D-glucopyranosyl-28-*O*-[*α*-L-rhamnosyl (1→2)-*β*-D-glucopyranosyl-16-deoxybarringtogenol C	Leaves	Xiao et al. (2013)
57	3-*O*-[*β*-D-glucopyranosyl (1→6)-(3′-*O*-angeloyl)-*β*-D-glucopyranosyl]-28-*O*-*β*-D-glucopyranosyl (1→6)[*α*-L-rhamnopyranosyl (1→2)-*β*-D-glucopyranosyl]16-deoxybarringtogenol C	Leaves	Xiao et al. (2013)
58	28*-O*-*β*-*D*-glucopyranosyl-16-deoxybarringtogenol C	Husks	Li (2006a)
59	3-*O*-[*β*-D-glucopyranosyl (1→6)] 3′- angeloyl)-*β*-D-glucopyranosyl-28-*O*-[*α*-L-rhamnosyl (1→2)]-*β*-D-glucopyranosyl-16-deoxybarringtogenol C	Carpophores	Li et al. (2008)
60	xanthohuskisides A	Husks	Li et al. (2013d)
61	xanthohuskisides B	Husks	Li et al. (2013d)
62	21*β*-*O*-acetylxanthohuskiside A	Husks	Wang et al. (2018)
63	3-*O*-*β*-D-glucopyranosyl-28-*O*-[*α*-L-rhamnopyranosyl (1→2)]-*β*-D-glucopyranosyl-16-deoxybarringtogenol C	Husks	Wang et al. (2016b)
64	*3- O*-[*β*-D-glucopyranosyl (1 → 6)]-[(3-*O*-angeloyl)-*β*-D-glucopyranosyl (1→2)]-*β*-D-glucopyranosyl-28-*O*-[*β*-D-glucopyranosyl (1→6)]-[*α*-L-rhamanopyranosyl (1→2)]-*β*-D-glucopyranosyl-16-deoxybarringtogenol C	Husks	Wang et al. (2016b)
65	3-*O*-[*β*-D-glucopyranosyl (1 → 6)]-(3-*O*-angeloyl) -*β*-D-glucopyranosyl-28-*O*-[*α*-L-rhamanopyranosyl (1→2)]-*β*-D-glucopyranosyl-16-deoxybarringtogenol C	Husks	Wang et al. (2016b)
66	sorbifoliaside	Husks	Fu et al. (2010)
67	xanifolia O_54_	Husks	Fu et al. (2010)
68	3-*O*-*β*-D-glucopyranosyl (1→6)-*β*-D-glucopyranosyl-28-*O*-*α*-L-rhamnopyranosyl (1→2)[*β*-D-glucopyranosyl (1→6)]*β*-D-glucopyranosyl-21*β*,22*α*-dihydroxyl-olean-12,15-diene	Husks	Li et al. (2013c)
69	3-*O*-*β*-D-glucopyranosyl (1→2)-*β*-D-glucopyranosyl-28-*O*-*α*-L-rhamnopyranosyl (1→2) [*β*-D-glucopyranosyl (1→6)]*β*-D-glucopyranosyl-21*β*,22*α*-dihydroxyl-olean-12-ene	Husks	Li et al. (2013c)
70	3-*O*-[α-L-arabinofuranosyl (1→3)]-[*β*-D-galactopyranosyl→2)]-*β*-D-(6-*O*-n-butyl)-glucuronopyranosyl-21-*O*-(3,4-*O*-diangeloyl)-*β*-D-fucopyranosyl-22-*O*-acetyl-barringtogenol	Husks	Wang et al. (2016b)
71	3-*O*-[*α*-L-arabinofuranosyl (1→3)]-[*β*-D-galactopyranosyl (1→2)]-*β*-D-(6-*O*-n-butyl)-glucuronopyranosyl-21-*O*-(3,4-*O*-diangeloyl)-*β*-D-fucopyranosyl-28-*O*-acetyl- barringtogenol C	Husks	Wang et al. (2016b)
72	3-*O*-[*α*-L-arabinofuranosyl (1 →3)]-[*β*-D-galactopyranosyl (1→2)]-*β*-D-(6-*O*-n-butyl)-glucuronopyranosyl-21,22-*O*-diangeloyl-R_1_-barrigenol C	Husks	Wang et al. (2016b)
73	3-*O*-[*α*-L-arabinofuranosyl (1→3)]-[*β*-D-galactopyranosyl (1→2)]-*β*-D-(6-*O*-n-butyl)-glucuronopyranosyl-21-*O*-angeloyl-22-*O*-(2-methyl)butyryl-R_1_-barrigenol C	Husks	Wang et al. (2016b)
74	3-*O-*[*α*-L-arabinofuranosyl (1→3)]-[*β*-D-glucopyranosyl (1→2)]-*β*-D-(6-*O*-n-butyl)-glucuronopyranosyl-21,22-*O*-diangeloyl-24-hydroxy-R_1_-barrigenol	Husks	Wang et al. (2016b)
75	*3- O*-[*α*-L-arabinofuranosyl (1 → 3)]-[*β*-D-galactopyranosyl (1→2)]-*β*-D-(6-*O*-n-butyl)-glucuronopyranosyl-21,22-*O*-diangeloyl-*β*arringtogenol C	Husks	Wang et al. (2016b)
76	napoleogenin B	Husks/Carpophores	Chen et al. (1985b)
77	22-*O*-acnapoleogenin B	Husks/Carpophores	Chen et al. (1985b)
78	21-*O*-(3,4-di-*O*-angeloyl)-*β*-D-fucopyranosyl theasapogenol B	Husks/Carpophores	Chen et al. (1985a)
79	21-*O*-(4-*O*-acetyl-3-*O*-angeloyl)-*β*-D-fucopyranosyl theasapogenol B	Husks/Carpophores	Chen et al. (1985a)
80	21-*O*-(4-*O*-acetyl-3-*O*-angeloyl)-*β*-D-fucopyranosyl-22-*O*-acetyl protoaescigenin	Husks/Carpophores	Chen et al. (1985a)
81	3-*O*-[*α*-L-arabinofuranosyl (1→3)]-*β*-D-galactopyranosyl (1→2)-*β*-D-6′-n-butyl-glucuronic acid-21-*O*-epoxyangeloyl-22-*O*-angeloyl-3*β*,16*α*,21*β*,22*α*,28-pentahydroxyolean-12-ene	Husks	Wang et al. (2016a)
82	16-*O*-acetyl-aesculioside G_12_	Husks	Wang et al. (2016a)
83	*3- O*-[*α*-L-arabinofuranosyl (1→3)]-*β*-D-galactopyranosyl (1→2)-*β*-D-6′-methyl-glucuronic acid-21-*O*-(3‴,4‴-*O*-diangeloyl)-*β*-D-fucopyranosyl-28-*O*-acetyl-3*β*,16*α*,21*β,*22*α*,28-pentahydroxy-olean-12-ene	Husks	Wang et al. (2016a)
84	6′-methylester-*O*-xanifolia-Y_8_	Husks	Wang et al. (2016a)
85	bunkankasaponin A	Seed oil residue	Yu et al. (2012a)
86	bunkankasaponin B	Seed oil residue	Yu et al. (2012a)
87	bunkankasaponin C	Seed oil residue	Yu et al. (2012a)
88	bunkankasaponin D	Seed oil residue	Yu et al. (2012a)
89	bunkankasaponin F	Seed oil residue	Yu et al. (2012a)
90	3-*O*-*β*-D-glucuronopyranoside bunkanka saponin A	Husks/Carpophores	Chen et al. (1985c)
91	16-*O*-acetyl-21-*O*-(3,4-di-*O*-angeloyl)-*β*-D*-*fucopyranosyl protoaescigen	Husks/Carpophores	Chen et al. (1985c)
92	oleanolic acid	Husks/Carpophores/Trunks and branches	Ma et al. (2000)
93	*β*-arabinopyranosyl-(1→4)-[*O*-*β*-D-galactopyranosyl-(1→6)-*O*-*β*-D-glucopyranosyl-(1→3)]-*O*-*β*-D-glucopyranosyluronic acid-(1→3)-gypsogenin	Husks/Carpophores	Chirva and Kintya (1971)
94	3-*O*-[*β*-D-galactopyranosyl (1→2)-*α*-L-arabinofuranosyl-(1→3)-*β*-D-methyl glucuronic acid 21-*O*-(3,4-diangeloyl)-*α*-L-rhamnose-3*β*, 16*α*,21*β*,22*α*,28*β*-pentahydroxyl-22-acetoxy-olean-12-ene	Husks	Guo et al. (2009)
95	sorbifoliasides K	Seed oil residue	Yu et al. (2012b)
96	3*β*,23-dihydroxy-lup-20 (29)-en-28-oic acid-23-caffeate	Husks/Carpophores	Li et al. (2007d)
97	3*β*,23-dihydroxy-lup-20 (29)-en-28-oic acid-3-caffeate	Husks/Carpophores	Li and Li (2008a)
98	betulin	Husks/Carpophore/Flowers	Li et al. (2005a); Zhao et al. (2012)
99	23-hydroxybetulinic acid	Husks/Carpophores	Li and Li (2008a)
100	3*α*,29-dihydroxytirucalla-7, 24-dien-21-oic acid	Husks/Carpophores	Ma et al. (2000)
101	3*β*-hydroxytirucalla-7, 24-dien-21-oic acid	Trunks and branches	Ma et al. (2000)
102	29-hydroxy-3-oxotirucalla-7,24-dien-21-oic acid	Trunks and branches	Ma et al. (2000)
103	29-*O*-acetyl-3-oxotirucalla-7,24-dien-21-oic acid	Trunks and branches	Ma et al. (2000)
104	3*β*,29-dihydroxytirucalla-7, 24-dien-21-oic acid	Trunks and branches	Ma et al. (2000)
105	3-oxotriucalla-7, 24-dien-21-oic acid	Trunks and branches	Ma et al. (2000)
106	24-methylenecycloartan-3-ol	Trunks and branches	Ma et al. (2000)
107	protoaescigenin	Husks/Carpophores	Chen et al. (1985b)
108	3-*O*-[*α*-L-arabinofuranosyl (1→3)]-[*β*-D-galactopyranosyl (1→2)]-*β*-D-6-*O*-methylglucuronopyranosyl-21*-O-*angeloyl-22-*O-*(2-methyl) butyryl-R1-barrigenol	Husks	Chen et al. (2020a)
109	3-*O*-*α*-D-glucopyranosyl-21,22-di-*O*-angeloyl-R1-barrigenol	Husks	Chen et al. (2020a)
110	3-*O*-*β*-D-6-*O*-methyl-glucuronopyranosyl-21-*O*-angeloy-22-*O*-isobutyryl-R1-barrigenol	Husks	Chen et al. (2020a)
111	21-acetyl-3-*O*-[*β*-D-glucopyranosyl (1→6)]- [angeloyl (1 → 3)]-*β*-D-glucopyranosyl-28-*O*-[*α-*L-rhamnopyranosyl (1→2)]-*β*-D-glucopyranosyl-16-deoxy-barringtogenol C	Husks	Chen et al. (2020a)
112	21-*O*-acetyl-3-*O*- [*β*-D-glucopyranosyl (1→6)]-[angeloyl (1→3)]-*β*-D-4-*O*-acetyl-glucopyranosyl-28-*O*-[*α*-L-rhamnopyranosyl (1→2)]-*β*-D-glucopyranosyl-16- deoxybarringtogenol C	Husks	Chen et al. (2020a)
113	21-acetyl-3-*O*-[*β*-D-glucopyranosyl (1→6)]-[angeloyl (1→ 4)]-*β*-D-glucopyranosyl-28-*O*-[*α*-L-rhamnopyranosyl (1→2)]-*β*-D-glucopyranosyl-16-deoxy-barringtogenol C	Husks	Chen et al. (2020a)
114	28-*O*-acetyl-21-*O*-*β*-D-fucopyranosyl barrigenol C	Husks	Chen et al. (2020a)
115	21-*O*-(3,4-di-*O*-angeloyl)-*β*-D-fucopyranosyl-R1-barrigenol	Husks	Chen et al. (2020a)
116	3-*O*-*α*-D-glucuronopyranosyl-21,22-di-*O*-angeloyl-R1-barrigenol	Husks	Chen et al. (2020a)
117	21-*O*-epoxyangeloyl-22-*O*-angeloyl-R1-barrigenol	Husks	Chen et al. (2020b)
118	21-*O*-(2, 3-di- hydroxy-2-methylbutyryl)-22-*O*-angeloyl-R1-barrigenol	Husks	Chen et al. (2020b)
119	28-*O*-isobutyryl-21-*O*-angeloyl-R1-barrigenol	Husks	Chen et al. (2020b)
120	3-*O*-*β*-D-6-*O*-methylglucuronopyranosyl-21-*O*-angeloyl-R1-barrigenol	Husks	Chen et al. (2020b)
121	3-*O*-*β*-D-6-*O*-methylglucuronopyranosyl-21-*O*-angeloyl-22-*O*-isobutyryl-R1-barrigenol	Husks	Chen et al. (2020b)
122	3-*O*-*β*-D-6-*O*-methylglucuronopyranosyl-21,22-di-*O*-angeloyl-R1-barrigenol	Husks	Chen et al. (2020b)
123	3-*O*-*β*-D-6-*O*-methylglucuronopyranosyl- 21-*O*-(3,4-di-*O*-angeloyl-*β*-D-fucopyranosyl) barrigenol C	Husks	Chen et al. (2020b)
124	3-*O*-[*β*-D-glucopyranosyl (1→6)]-(2-angeloyl)-*β*-D-glucopyranosyl-28-*O*-*β*-D-glucopyranosyl (1→6)[*α*-L-rhamnopyranosyl (1→2)-*β*-D-glucopyranosyl]-21-*O*-acetyl-16-deoxybarringtogenol C	Husks	Ding et al. (2019)
**Flavonoids**			
125	quercetin	Trunks and branches/Leaves/Husks/Flowers	Zhang and Bao (2000); Wu (2017); Zhao et al. (2012)
126	myricetin	Trunks and branches/Leaves/Husks	Zhang and Bao (2000); Wu (2017)
127	kaempferol	Trunks and branches/Leaves/Husks/Flowers	Li (2006a); Zhao et al. (2012)
128	rhamnocitrin	Trunks and branches/Leaves/Husks/Flowers	Zhao et al. (2012)
129	mearnsetin	Husks	Manthey and Guthrie (2002)
130	quercimetrin	Husks	Panyadee et al. (2015)
131	quercitrin	Flowers	Zhao et al. (2012)
132	isoquercitrin	Trunks and branches/Leaves/Husks	Zhao et al. (2013); Aderogba et al. (2013)
133	3-*O*-methyl-quercetin	Trunks and branches/Leaves/Husks	Zhao et al. (2013)
134	myricitrin	Trunks and branches/Leaves/Husks	Kang et al. (2012); Wu (2017)
135	isomericitrin	Husks/Flowers	Yang et al. (2016a); Zhao et al. (2012)
136	kaempferol-3-*O*-*α*-L*-*rhamnopyranoside	Trunks and branches/Leaves/Husks	Zhao et al. (2013)
137	kaempferol-3-*O-β*-D-glucopyranoside	Trunks and branches/Leaves/Husks/Flowers	Zhao et al. (2013)
138	kaempferol-3-*O*-(2-*O-α*-L-rhamnopyranosyl)-glucopyranoside	Trunks and branches/Leaves/Husks	Zhao et al. (2012)
139	kaempferol-3-*O*-rutinoside	Trunks and branches/Leaves/Husks/Flowers	Li (2006a); Zhao et al. (2012); Yang et al. (2016a)
140	rutin	Husks	Li (2006a)
141	chrysoeriol	Trunks and branches/Leaves/Husks/Flowers	Zhao et al. (2012)
142	tricetin	Husks	Yang et al. (2016a)
143	myricetin 3-*O*-rutinoside	Husks	Yang et al. (2016a)
144	isorhamnetin 3-*O*-rutinoside	Husks	Yang et al. (2016a)
145	luteolin	Husks	Wan et al. (2015)
146	naringenin	Trunks and branches/Leaves/Husks/Flowers	Wu (2017); Zhao et al. (2012); Yang et al. (2016a)
147	eriodictyol	Trunks and branches/Leaves/Husks	Li (2006a); Li et al. (2006b); Yang et al. (2016a)
148	eriodictyol 4′-*O-β*-D-glucopyranoside	Husks	Yang et al. (2016a)
149	(2S)-eriodictyol-7-*O-β*-D-glucopyranoside	Husks	Yang et al. (2016a)
150	(2R)-eriodictyol-7-*O-β*-D-glucopyranoside	Husks	Yang et al. (2016a)
151	naringenin 5-*O-β*-D-glucopyranoside	Husks	Yang et al. (2016a)
152	naringenin 4′-*O-β*-D-glucopyranoside	Husks	Yang et al. (2016a)
153	(-)-salipurposide	Husks	Yang et al. (2016a)
154	naringenin-7-*O-β*-D-glucopyranoside	Husks	Wan et al. (2015)
155	2*α*-3′,4′,5,5′,7-pentahydroxyflavone	Trunks and branches	Wu (2017)
156	3,3′,4′,5,7-pentahydroxyflavanone	Trunks and branches	Wu (2017)
157	2*β*,3*β*-3,3′,5,5′-pentahydroxyflavone	Trunks and branches	Wu (2017)
158	2*α*, 3*β*-dihydroquercetin	Trunks and branches/Leaves/Fruits	Zhang and Bao (2000); Wu (2017)
159	dihydromyricetin	Trunks and branches/Leaves/Husks	Zhang and Bao, 2000; Wu (2017)
160	aromadendrin	Husks	Yang et al. (2016a)
161	taxifolin	Husks	Yang et al. (2016a)
162	catechin	Husks	Yang et al. (2016a)
163	gallocatechin	Trunks and branches/Husks	Ni and Zhang (2009); Yang et al. (2016a)
164	(−)-epicatechin	Trunks and branches/Leaves/Husks	Zhang and Bao (2000)
165	(−)-epigallocatechin	Trunks and branches/Leaves/Husks	Huang and Feng (1987)
166	(−)-epiafzelechin	Trunks and branches/Leaves/Husks	Ma and Nakamura (2004)
167	epicatechin-5-*O-β*-D-glucopyranaoside	Seed oil residue	Yu et al. (2018)
168	epigallocatechin-(4*β*→8,2*β*→*O*-7)-epicatechin	Trunks and branches	Wu (2017)
169	procyanidin A-2	Trunks and branches	Wu (2017)
170	proanthocyanidin A2	Trunks and branches	Wu (2017)
171	cirmamtanninB-1	Husks	Yang et al. (2016a)
172	2*α*,3*α*-epoxy-5,7,3′,4′-tetrahydroxyflavan-(4*β*-8-catechin)	Trunks and branches	Wu (2017)
**Phenylpropanoids**			
173	fraxin	Seed oil leavings	Zhu et al. (2018)
174	fraxetin	Seed oil residue	Chen et al. (1984)
175	isofraxetin	Fruits	Li (2006a)
176	isofraxetin-6-*O*-*β*-D-glucopyranoside	Husks	Li (2006a)
177	fraxetin-7-*O*-*β*-D-[6’-(3″-hydroxyl-3‴- methylglutaryl)] glucopyranoside	Seed oil residue	Zhu et al. (2018)
178	scopoletin	Seed oil residue	Zhu et al. (2018)
179	isoscopoletin	Flowers	Zhao et al. (2013)
180	esculetin	Seed oil residue	Zhu et al. (2018)
181	xanthotoxin	Husks	Wan et al. (2015)
182	cleomiscosin D	Husks	Li (2006a); Li et al. (2006b)
183	cleomiscosin B	Husks	Li et al. (2007b)
184	meso-2,3-di (3′,4′-methylenedioxybenzyl) butane-1,4-diol	Seed oil residue	Yu et al. (2018)
185	umbelliferone	Husks	Yang et al. (2020a)
186	biscopoletin	Husks	Yang et al. (2020a)
**Steroids**			
187	22, 23-dehydroxy-chondeillasterone	Husks/Carpophores	Wan et al. (2013)
188	stigmasterol acetate	Husks	Li et al. (2007a)
189	(3*β*,5*α*,20*R*,24*S*)-stigmasta-7,trans-22-dien-3-ol	Husks	Cheng et al. (2001)
190	(3*β*,5*α*,20*R*,24*S*)-stigmasta-7-en-3-ol	Husks	Cheng et al. (2001)
191	*α*-spinasterol	Husks	Li et al. (2005a)
192	ergosterol peroxide	Husks	Li (2006a)
193	9 (11)-dehydro-ergosterol peroxide	Husks	Li (2006a)
194	5*α*,8*α*-epidioxy-(22*E*,24*R*)-ergosta-6,22-dien-3*β*-ol	Husks	Li (2006a)
195	Δ^7^-stigmastenol	Carpophores	Li et al. (2005a)
196	Δ^7^-avenasterol	Carpophores	Li et al. (2005a)
197	daucosterol	Carpophores	Li et al. (2005a)
198	*β*-sitosterol	Trunks and branches/seed oil residue	Li et al. (2005a);Yu et al. (2018)
199	stigmasterol	Trunks and branches/seed oil residue	Dong et al. (2008); Li et al. (2007a)
200	*β*-sitosterol acetate	Kernel oil	Yan et al. (1984)
201	campesterol	Kernel oil	Yan et al. (1984)
202	campesterol acetate	Kernel oil	Yan et al. (1984)
203	cholesterol	Kernel oil	Yan et al. (1984)
**Phenols**			
204	2-hydroxy-6-methylbenzoic acid	Trunks and branches/Husks	Li (2006a); Wu (2017)
205	vanillic acid	Husks	Li (2006a)
206	gallic acid	Husks	Yang et al. (2016a)
207	tyrosol	Husks	Li (2006a)
208	4-hydroxyphenylacetic acid	Husks	Li (2006a)
209	protocatechuic acid	Husks	Yang et al. (2016a)
210	isochlorogenic acid B	Trunks and branches	Wu (2017)
211	4-hydroxybenzaldehyde	Husks	Li (2006a)
212	*p*-hydroxybenzoic acid	Husks	Yang et al. (2016a)
213	xspolyphenol A	Husks	Yang et al. (2016a)
214	xspolyphenol B	Husks	Yang et al. (2016a)
215	pyrogallol	Husks	Yang et al. (2016a)
216	hydroquinone	Husks	Wan et al. (2015)
217	4-hydroxybenzylcyanide	Husks	Wan et al. (2015)
218	5,7-dihydroxychromone	Trunks and branches/Leave/Husk	Li (2006a)
219	methyl 4-hydroxylbenzoate	Husks	Yang et al. (2016a)
220	methyl caffeoate	Seed oil residue	Yu et al. (2018)
**Fatty acids**			
221	nonadecanoic acid	Trunks and branches	Li et al. (2007a)
222	heneiosanoic acid	Trunks and branches	Li et al. (2007a)
223	tetracosanoic acid	Trunks and branches	Li et al. (2007a)
224	myristic acid	Kernels	Liang et al. (2021)
225	palmitoleic acid	Kernels	Liang et al. (2021)
226	arachidic acid	Kernels	Liang et al. (2021)
227	docosadienoic acid	Kernels	Liang et al. (2021)
228	tricosanoic acid	Kernels	Liang et al. (2021)
229	nervonic acid	Kernels	Liang et al. (2021)
230	oleic acid	Kernels	Wang (1998)
231	erucic acid	Kernels	Wang (1998)
232	linolenic acid	Kernels	Wang (1998)
233	eicosadienoic acid	Kernels	Wang (1998)
234	stearic acid	Kernels	Wang (1998)
235	eicosanoic acid	Kernels	Wang (1998)
236	lignoceric acid	Kernels	Wang (1998)
237	behenic acid	Kernels	Wang (1998)
238	dodecanoic acid	Kernels	Wang (1998)
239	hexanoic acid	Husks	Cheng et al. (2002)
240	heptanoic acid	Husks	Cheng et al. (2002)
241	nonanoic acid	Husks	Cheng et al. (2002)
242	decanoic acid	Husks	Cheng et al. (2002)
243	10-methylundecanoic acid	Husks	Cheng et al. (2002)
244	12-methyltetradecanoic acid	Husks	Cheng et al. (2002)
245	heptadecanoic acid	Husks	Cheng et al. (2002)
246	palmitic acid	Kernels oil	Bao et al. (2012)
247	9,12-octadecadienoic acid	Kernels oil	Li et al. (2013a)
248	9-octadecadienoic acid	Kernels oil	Li et al. (2013a)
249	11-eicosenoic acid	Kernels oil	Li et al. (2013a)
**Alkaloids**			
250	2-methyl-6-(2′, 3′, 4′-trihydroxybutyl) -pyrazine	Husks	Li (2006a); Li et al. (2006b)
251	indole-3-carboxaldehyde	Seed oil residue	Yu et al. (2018)
252	allantoin	Seed oil residue	Yu et al. (2018)
253	indole-3-acetylaspartic acid	Seed oil residue	Yu et al. (2018)
254	1-oxa-2-azaspiro [4.5]dec-2-ene-8-ol	Husks	Ge et al. (2016)
255	*trans-*xanthoisoxazoline A	Flowers/Husks	Li et al. (2018)
256	*cis-*xanthoisoxazoline A	Flowers/Husks	Li et al. (2018)
257	xanthoisoxazoline B	Flowers/Husks	Li et al. (2018)
258	xanthoisoxazoline C	Flowers/Husks	Li et al. (2018)
**Quinones**			
259	2,5-dimethoxy-p-benzoquinone	Trunks and branches	Dong et al. (2008); Ni and Zhang (2009)
260	physcion	Trunks and branches	Dong et al. (2008)
261	chrysophanol	Fruits	Dong et al. (2008)
262	emodin	Fruits	Dong et al. (2008)
**Others**			
263	3,4,5-trimethoxy benzoic acid	Trunks and branches	Li (2006a)
264	succinic acid	Husks	Chen et al. (1984)
265	4-(*α*-hydroxyethyl)cyclohexan-1-oic acid	Husks	Yang et al. (2020a)
266	vomifoliol-3′-*O*-*β*-D-apiofuranosyl-(1–6)-*β*-D-glucopyranoside	Seed oil residue	Yu et al. (2018)
267	dihydrophaseic acid 3′-O-*β*-D-glucopyranoside	Seed oil residue	Yu et al. (2018)
268	xanthocerapene	Trunks and branches	Wu (2017)
269	1,4-di-(2-cyanostyryl)benzene	Husks	Li (2006a)
270	1-*O*-methyl-myo-inositol	Flowers	Zhao et al. (2012)
271	ethyl nonanoate	Husks	Yang et al. (2020a)
272	methyl (2*E*,5*S*)-(-)-5-methyldodec-2-enoate	Husks	Li (2006a)
273	heptadecan-1-ol	Husks	Yang et al. (2020a)
274	pentadecanoic acid heptadecyl ester	Husks	Yang et al. (2020a)
275	bungeinA	Husks	Yang et al. (2020a)
276	tocopherols	Kernels	Liang et al. (2021)
277	vitamin A	Kernels	Liang et al. (2021)
278	vitamin C	Kernels	Liang et al. (2021)

### Triterpenoids

Triterpenoids represent a large part of the chemical constituents in the *X. sorbifolium*, with 124 triterpenoid compounds having been identified from the husks, carpophores, leaves, and seeds (compounds **1–124**, Figure 2). Yu et al. (2012a) extracted the seed oil residue of *X. sorbifolium*. The compounds were separated by D-101 macroporous resin, silica gel column chromatography, Sephadex LH-20, octadecylsilyl (ODS) column, and purified by prep-HPLC chromatography. Seven new oleanane-type triterpenoid saponins, sorbifoliaside A-J (**35–44**), were identified by MS, ^1^H-NMR, ^13^C-NMR, ^1^H-^1^H COSY, HSQC, HMBC, NOESY, and TOCSY methods (Yu et al., 2012a; Yu et al., 2012b). Wang et al. (2016b) extracted *X. sorbifolium* with ethanol, analyzed the compounds by Sephadex LH-20, ODS, UV, MS, and NMR, and identified triterpenoids: 3-*O-β-*D-glucopyranosyl-28-*O*-[*α*-L-rhamnopyranosyl (1→2)]-*β*-D-glucopyranosyl-16-deoxybarringtogenol C **(63)**, 3-*O*-[*β*-D-glucopyranosyl (1→6)]-[(3-*O*-angeloyl)-*β*-D-glucopyranosyl (1→2)]-*β*-D-glucopyranosyl-28-*O*-[*β*-D-glucopyranosyl (1→6)]-[*α*-L-rhamanopyranosyl (1→2)]-*β*-D-glucopyranosyl-16-deoxybarringtogenol C **(64)**,3-*O*-[*β*-D-glucopyranosyl (1 →6)]-(3-*O*-angeloyl)-*β*-D-glucopyranosyl-28-*O*-[*α*-L-rhamanopyranosyl (1→2)]-*β*-D-glucopyranosyl-16-deoxybarringtogenol C **(65)** (Wang et al., 2016b). Chen et al. (2020a) extracted 70% ethanol from the husk of *X. sorbifolium*; separated and identified a series of compounds by D-101 macroporous resin, silica gel column, ODS column, and HPLC chromatography; and isolated compounds **108–123** for the first time (Chen et al., 2020a; Chen et al., 2020b). The chemical structures of triterpenoids are provided in Figure 2.

### Flavonoids

Flavonoids are a group of naturally occurring compounds that contain a benzopyran heterocycle linked to a benzene ring (Testai, 2015). Currently, 48 flavonoids (**125–172**) have been obtained from the trunks and branches, leaves, husks, and flowers of *X. sorbifolium.* Among these compounds, quercetin and myricetin are the main aglycons. Zhang and Bao (2000) used polyamide and silica gel column chromatography to isolate the chemical constituents of lignum xanthocerais. Two flavonoids, 2*α*, 3*β*-dihydroquercetin **(158)**, epicatechin **(163)**, were identified by UV, MS, ^1^H-NMR, ^13^C-NMR, and 2D-NMR (Zhang and Bao, 2000). Wu (2017) separated and purified the acetone extract of lignum xanthocerais by ODS, Sephadex LH-20, and preparative high-performance liquid chromatography (HPLC). After that, eight flavonoids, namely myricitrin **(134)**, rutin **(140)**, 3, 3′, 4′, 5, 7-pentahydroxyflavanone **(156)**, dihydromyricetin **(159)**, catechin **(161)**, gallocatechin **(162)**, epigallocatechin **(165)**, procyanidin A-2 **(169),** were identified by Thin layer chromatography (TLC), ^1^H-NMR, ^13^C-NMR, and MS (Wu, 2017). The chemical structures of flavonoids are provided in Figure 3.

### Phenylpropanoids

Phenylpropanoids are natural compounds with benzene rings. Phenylpropanoids generally contain a phenol structure and are a phenolic substance. Fourteen simple phenylpropanoids have been extracted from *X. sorbifolium*, with their main components being coumarins (compounds **173–181, 185–186**) and lignans (compounds **182–184**). Zhu et al. (2018) isolated and purified the chemical composition of seed oil residue of *X. sorbifolium* by silicone, macroporous, Sephadex LH-20, and ODS column chromatography. Four phenylpropanoid compounds, namely fraxin **(173)**, fraxetin-7-*O*-*β*-D-[6’-(3″-hydroxyl-3‴- methylglutaryl)] glucopyranoside **(177)**, scopoletin **(178)**, and esculetin **(180)** were identified by spectral and chemical methods (Zhu et al., 2018). The chemical structures of the phenylpropanoids are provided in Figure 4.

### Steroids

Steroids are present in almost all plants and exhibit significant biological activity. Phytosterol is a steroid derivative of the C_17_ side chain with 8–10 carbon atoms in the side chain. At present, 17 steroids (compounds **187–203**) have been reported from the wood, husks, carpophores, seed oil residue, and kernel oil of *X. sorbifolium*. Yan et al. (1984) used TLC, impregnated silica gel G with 18% silver nitrate, and separated kernel oil of *X. sorbifolium* with petroleum ether (7:3, V/V) as the developing agent. The steroid compounds *β*-sitosterol acetate **(200)**, campesterol **(201)**, campesterol acetate **(202)**, and cholesterol **(203)** were identified by TLC, MS, FT-IR, and GC-MS (Yan et al., 1984). Cheng et al. (2001) separated the husk of *X. sorbifolium* by column chromatography and spectroscopy to obtain two steroids (3*β*,5*α*,20*R*,24*S*)-stigmasta-7,trans-22-dien-3-ol **(189)** and (3*β*,5*α*,20*R*,24*S*)-stigmasta-7-en-3-ol **(190)** (Cheng et al., 2001). The chemical structures of the steroids are provided in Figure 5.

### Phenols

Through this review, 17 phenolics (compounds **204–220**) were found in *X. sorbifolium*. Wan et al. (2015) studied the chemical constituents of the husk of *X. sorbifolium*. They were isolated and purified by TLC, Sephadex LH-20 column, ODS column, and preparative HPLC. Two phenolic compounds, hydroquinone **(216)** and 4-hydroxybenzylcyanide **(217)** were identified based on physicochemical properties and spectral data (Wan et al., 2015). Wu (2017) separated and purified the acetone extract of lignum xanthocerais by ODS, Sephadex LH-20, and preparative HPLC. After that, two phenolic compounds, protocatechuic acid **(209)** and isochlorogenic acid B **(210)**, were identified by TLC, ^1^H-NMR, ^13^C-NMR, and MS (Wu, 2017). The chemical structures of the phenolic compounds are provided in Figure 6.

### Fatty Acids

The fatty acid components are concentrated in the kernels and husks of *X. sorbifolium*. At present, 29 kinds of fatty acids (compounds **221–249**) have been identified. Cheng et al. (2002) used GC-MS to separate and identify seven fatty acid compounds from the husk of *X. sorbifolium*, which were hexanoic acid **(239)**, heptanoic acid **(240)**, nonanoic acid **(241)**, decanoic acid **(242)**, 10-methylundecanoic acid **(243)**, 12-methyltetradecanoic acid **(244)** and heptadecanoic acid **(245)** (Cheng et al., 2002). The chemical structures of fatty acids are provided in Figure 7.

### Alkaloids

Nine alkaloids (compounds **250–258**) were obtained from the methanol extract of the seed and husks. Among these compounds, Yu et al. (2018) identified the chemical constituents of lignum xanthocerais by 1D- and 2D-NMR, and ESI-MS and obtained three alkaloid compounds, indole-3-carboxaldehyde (**251)**, allantoin **(252)**, indole-3-acetylaspartic acid **(253)** (Yu et al., 2018). The chemical structures of alkaloids are provided in Figure 8.

### Quinones

Four quinones (compounds **259–262**) were also found in the fruits and wood of *X. sorbifolium*. Dong et al. (2008) used silica gel column, preparative TLC, and pharmadex LH-20 column chromatography to isolate compounds from the fruits of *X. sorbifolium* and identified their structure by a spectral method. As a result, four quinone compounds were isolated: 2,5-dimethoxy-p-benzoquinone **(259)**, physicone **(260),** chrysophanol **(261)**, and emodin **(262)** (Dong et al., 2008). The chemical structures of quinones are provided in Figure 9.

### Others

In addition to the constituents mentioned previously, an additional sixteen compounds (**263–278**) were identified. Moreover, some nutritional elements were also found to be abundant in the herb. More than ten amino acids were found in the seeds of *X. sorbifolium*. It is worth noting that the predominant amino acids present are glutamic plus glutamine, aspartic plus asparagine, and arginine. These amino acids account for up to 43% of the total amino acids present in the species (Fan et al., 2009; Mónica et al., 2017). The chemical structures of other compounds are provided in Figure 10.

## Pharmacological Activities

The pharmacological properties of *X. sorbifolium* have attracted a great deal of attention in recent years. The main pharmacological activities of *X. sorbifolium* include improving learning and memory impairments, anti-inflammatory, anti-tumor, and anti-oxidation. In particular, the triterpene saponin xanthoceraside, a characteristic compound of *X. sorbifolium*, shows excellent learning and memory improvement, anti-inflammatory, and anti-tumor activities. Table 2 lists some *in vitro* and *in vivo* pharmacological models and related dosage information to clarify the pharmacological activities of *X. sorbifolium.*

**TABLE 2:** The pharmaceutical effects of *X. sorbifolium.*

Pharmaceutical effects	Used part	Compounds/extracts	Doses	Models	Results/mechanism	References
Improving learning and memory impairments	Fruit stalks	3-*O*-[*β*-D-glucopyranosyl (1 → 6)] (3′-*O*-angeloyl)-*β*-D-glucopyranosyl, 28-*O*-[*β*-D-glucopyranosyl (1 → 6)]-[*α*-L-rhamnopyranosyl (1 → 2)]-*β*-D-glucopyranosyl 16-deoxybarringtogenol C	0.32 mg kg^−1^	Male Kunming (KM) mice	Significantly protective against ICV-Aβ_1-42_. induced learning and memory impairment	Li et al. (2020)
	Husks	Xanthoceraside	0.02, 0.08 and 0.32 mg kg^−1^	Male ICR mice	Xanthoceraside inhibition of the TLR2 pathway and down-regulation of MAPK and NF-κB activities may be related to the improvement in learning and memory impairments	Qi et al. (2017)
	Husks	Xanthoceraside	0.056, 0.112, and 0.224 mg kg^−1^	Male Sprague-Dawley (SD) rats	Xanthoceraside can modulate the structure of gut microbiota in AD rats, and the gut microbiota may be potential targeting territory of xanthoceraside via microbiome-gut-brain pathway	Zhou et al. (2019)
	Husks	Xanthoceraside	0.01, 0.05 and 0.1 mg mL^−1^	SD rats	Exhibited obvious neuroprotection against amyloid-β-induced cytotoxicity on PC12 cells, indicating their potential to be bioactive substances against Alzheimer’s disease	Rong et al. (2018)
	Husks	Xanthoceraside	0.06, 0.12, and 0.24 mg kg^−1^	Rats	Both memory deficits and insulin receptor and insulin-like growth factor-I receptor (IGF-1R) protein expression levels were ameliorated	Liu et al. (2013)
	Husks	Xanthoceraside	0.01 and 0.1 μM	SH-SY5Y cells	Significantly increased the survival rate of SH-SY5Y cells injured by Aβ_25-35_ in a dose-dependent manner	Chi et al. (2013)
	Husks	Total triterpenoid saponins	0.93, 2.8, and 8.4 mg kg^−1^	KM mice and SD rats	Significantly improve the impairments of learning and memory. The preliminary mechanism might associate with its protection effects against oxidative stress damage, cholinergic system deficiency and synaptic damage	Ji et al. (2017)
	Husks	Crude extract of the husks	50 mg kg^−1^	SD rat	Crude extract of the husks from Xanthoceras sorbifolia might ameliorate the impairment of learning and memory in the Alzheimer’s disease animal model with similar function of AchEI as huperzine	Sun et al. (2018)
	Husks	Ethanol extracts	89.80, 44.90 mg kg^−1^	KM mice	It significantly improved the impairment of learning and memory, and the mechanism may be related to the enhancement of the function of the central cholinergic and glutamatergic nervous systems, and resistance to oxygen consumption injury in brain tissue	Liu et al. (2007b)
	Husks	Ethanol extracts	2.5, 5.0, and 10.0 mg kg^−1^·D^−1^	AD rat model	Oral treatment with XSE significantly reduced cognitive impairments in behavioral tests (passive avoidance test, novel object recognition test, Y-maze test and Morris water maze test). The cognition-improving effects of XSE probably resulted from dendritic spine protection effects through regulation of BDNF signaling pathways.	Li et al. (2016b)
	Husks	Ethanol extract	62.9 mg⋅kg^−1^	Wistar rats	Significantly improve the learning and memory ability of rats, increase superoxide dismutase activity, reduce MDA and acetylcholinesterase levels, and significantly inhibit the degeneration and shedding of hippocampal neurons	Liu et al. (2007a)
Anti-inflammatory	Leaves	Quercetin-3-*O-β*-D-glucopyarnoside, catechin, syringaresinol-4-*O-β*-D-glucopyranoside, 4-*O-β*-D-glucopyranosyl-trans-*p*-coumaric acid	IC_50_ 13.39 ± 1.27 µM, 9.52 ± 2.18 µM, 3.08 ± 1.77 µM, and 9.08 ± 1.23 µM	LPS-induced BV2 cells	The compounds exhibited much stronger inhibiting effect on NO production than that of the positive control minocycline (IC_50_ 37.04 ± 2.09 µM) in LPS-induced BV2 cells	Li et al. (2016a)
	Husks	Xanthoceraside	0.01 and 0.1 μM	Microglial cells	The inhibitory effect of xanthoceraside on pro-inflammatory mediators was possibly mediated through TLR2 receptor/MyD88 in A*β*_25-35_/IFN-g-stimulated N9 microglial cells	Qi et al. (2013)
	Wood	n-Butanol extract	1.0, 2.0 g kg^−1^	Wistar rats	It has antagonistic effect on adjuvant arthritis in rats, and its mechanism may be related to the inhibition of immune function	Kuang and Liu (2002)
Anti-tumor	Husks	Xanifolia Y	2.5, 5, and 10 mM	HepG2, HCT116, and U87-MG cell lines	It could suppress U87-MG cell proliferation by inducing apoptosis in the early period of exposure and then promote arrest at the G0/G1 phase	Wang et al. (2016a)
	Husks	Xanthoceraside	10 μM	A375.S2 cells	Xanthoceraside selectively inhibits the proliferation of human melanoma A375 cell line and induces apoptosis through the mitochondria-mediated apoptotic pathway	Jiao et al. (2014)
	Husks	3-*O*-[*α*-L-arabinofuranosyl (1→3)]-[*β*-D-galactopyranosyl (1→2)]-*β*-D-(6-*O*-n-butyl)- glucuronopyranosyl-21, 22-*O*-diangeloyl-R_1_-barrigenol	9.75 and 17.32 μM	HepG2, HCT-116 cell	The compound showed significant inhibitory activity against the proliferation of HepG2, HCT-116 cell lines	Wang et al. (2016b)
	Wood	3-Oxotirucalla-7,24-dien-21-oic acid, oleanolic acid, epicatechin	20, 10, and 70 μg mL^−1^	HIV-1 PR	They were found to be inhibitory substances against human immunodeficiency virus (HIV-1) protease	Ma et al. (2000)
	Kernels	Total saponins	9.7 ± 0.39 mg L^−1^	HepG2 cell	Total saponins can induce apoptosis of HepG2 cells. The flow cytometry showed that the late apoptosis of cancer cells may be concentrated in the S phase of cell cycle	Zhang et al. (2016)
	Husks	95% Ethanol-water extract	75 μg mL^−1^	HepG2 cell	When the mass concentration of 95% ethanol water extract was 75 μg mL^−1^, the inhibition of HepG2 cell proliferation effect was up to 70.1%	Zhang et al. (2017)
Anti-oxidation	Wood	Catechin, epicatechin, myricetin, and dihydromyricetin	6.5, 4.2, 3.8, and 5.7 μg mL^−1^	-	These four compounds has been shown to scavenge DPPH radicals, with EC_50_ values of 6.5, 4.2, 3.8, and 5.7 μg mL^−1^, and afford remarkable protection of peroxyl radical-induced DNA strand scission, exhibiting protection values of 92.10, 94.66, 75.44, and 89.95% at a concentration of 10 μmol L^−1^	Zhang et al. (2015)
	Husks	Total saponins	50 μg mL^−1^	A549, HepG2, MGC-803, and MFC cell lines	The total saponins have shown the ability to scavenge hydroxyl free radicals and superoxide anion free radicals; this scavenging ability exhibited a dose-effect relationship with concentration	Yang et al. (2016a)
	Seeds	Seed oil	0.151, 0.195 g mL^−1^	-	The seed oil exhibited notable DPPH radical-scavenging activity and lipid peroxidation inhibitory activity with IC_50_ values of 0.151 and 0.195 g mL^−1^	Zhang et al. (2010)
	Husks	Saponins	0.18–2.52 mg mL^−1^	The rate of tyrosinase catalyzed L-DOPA oxidation	At a concentration of 0.18–2.52 mg mL^−1^, the hydroxyl radical-scavenging effect of the saponins form *X. sorbifolium* husks was 15.5–68.7%	Zhang and Zhou (2013a)
	Kernels	Microwave-assisted extraction extract of triterpene saponins	0.782 mg mL^−1^	-	Microwave-assisted extraction extract of triterpene saponins exhibited substantial free radical-scavenging activity with an IC50 value of 0.782 mg mL^−1^	Li et al. (2010)
	Seeds	Seed oil	0.11, 0.22, 0.33 ml/(20 g bw)	Male KM mice	The activities of antioxidant enzymes such as SOD, CAT and GSH-Px in liver and brain of mice in the cold pressing oil test group were significantly higher than those in the normal control group	Deng et al. (2010)
	Seeds	Seed oil	0.1–1.4 g·mL^−1^	-	Seed oil has a good scavenging effect on hydroxyl radical and superoxide anion radical, has a strong scavenging effect on DPPH radical, and its reduction ability exceeds BHT and TBHQ. It also has a good inhibitory effect on Fe^2+^ induced lipid peroxidation at higher concentration	Deng et al. (2012)
	Husk	Ethanol extract	0.2 mg·mL^−1^	-	The ethanol extracts exhibited a scavenging effect on DPPH, with the 70% ethanol aqueous extract showing the strongest activity for scavenging the DPPH free-radical at a concentration of 0.2 mg·mL^−1^	Zhang et al. (2017)
Antidepressant	Husks	Xanthoceraside	0.02, 0.08 and 0.32 mg·kg^−1^	Adult male C57BL/6J mice	Xanthoceraside possesses antidepressant effects in mice which are mediated by activation of hippocampal BDNF signaling pathway	Guan et al. (2021)
Anti-HIV	Seed coat	Cleomiscosin B	8.61–12.76 μg·mL^−1^	C8166 cell	The cleomiscosin B have exhibited an effect on HIV-1 IIIB-induced C8166 cell formation in syncytia with an EC_50_ of 8.61–12.76 μg·mL^−1^, as well as a protective effect on MT4 cells infected by HIV-1 IIIB.	Li et al. (2007b)

### Improving Learning and Memory Impairments

Improving learning and memory impairments is mainly demonstrated through the regression of Alzheimer’s disease (AD). AD is a neurodegenerative disease that exhibits relentless progression in cognition impairment and memory dysfunction. Its formation and development are closely associated with the neurotoxicity of extracellular amyloid-beta (Aβ) deposits (Li et al., 2020). The specific mechanisms include the induction of apoptosis (Qu et al., 2000), activation of glial cells to induce inflammatory cascades (Li et al., 1998), triggering of oxidative stress (Huang et al., 1999), increase in intracellular Ca^2+^, and reduction of cell membrane fluidity (Tian et al., 2001). Among these, Aβ-associated oxidative stress and related antioxidant defense system deficits are fundamental mechanisms in AD etiopathogenesis (Ma and Klann, 2012).

Previous studies indicate that barrigenol-type triterpenoids exhibit remarkable protective effects against spatial memory impairments. As such, they have the potential to be used in AD therapy and other neurodegenerative diseases. For instance, Qi et al. (2017) used an intracerebroventricular injection of amyloid 1–42 (Aβ1-42) to establish a mouse model to test the effect of xanthoceraside on Aβ-induced cognitive dysfunction and the influence of the TLR2/NF-κB and MAPK pathway. The results showed that xanthoceraside at doses of 0.08 and 0.32 mg kg^−1^ significantly improved learning and memory impairments in mice and significantly inhibited Aβ1-42-induced overexpression of GFAP and CD11b. The results suggested that xanthoceraside inhibited the TLR2 pathway and downregulated MAPK and NF-κB activity, which may be associated with improved learning and memory impairment (Qi et al., 2017). Li et al. (2020) isolated 8 kinds of barrigenol-type triterpenoids, all of which firstly detected the oxidative stress effect of hydrogen peroxide on human SH-SY5Y cells. Then Y-maze, Morris water maze, new object recognition, and passive avoidance tests were used to evaluate the improvement effect of the selected compounds on ICV Aβ1-42 mice. The compounds, (3-*O*-[*β*-D-glucopyranosyl (1→6)] (3′-*O*-angeloyl)-*β*-D-glucopyranosyl and 28-*O*-[*β*-D-glucopyranosyl (1→6)]-[*α*-L-rhamnopyranosyl (1→2)]-*β*-D-glucopyranosyl 16-deoxybarring-togenol C (0.32 mg kg^−1^) showed significant improvements in enhancing memory disorders, object recognition defects, learning and memory impairments, and spatial memory disorders induced by Aβ_1-42_ (410 pmol in 3 μL) in intracerebroventricular (ICV)-injected mice (Li et al., 2020).

Ji et al. (2017) reported that total triterpenoid saponins from *X. sorbifolium* husks significantly improves learning and memory impairments. Specifically, it significantly increased spontaneous alternation in the Y maze test and prolonged swimming duration in the fourth quadrant of the Morris water maze probe test at a dosage of 8.4 mg kg^−1^. This substance also improved escape latency and passive avoidance test results in a dose-dependent manner. The primary mechanism might be associated with its protective effects against oxidative stress damage, cholinergic system deficiency, and synaptic damage (Ji et al., 2017). A study conducted using a rat AD model with ICV injection of Aβ_25–35_ revealed that rats receiving 70% aqueous ethanol extracts containing husks of *X. sorbifolium* (5 and 10 mg kg^−1^) demonstrated an upregulation of brain-derived neurotrophic factor (BDNF) expression, which protects the dendritic spine and achieves cognition-improving effects. The primary mechanism was a decrease in the dendritic spine density via activation of the BDNF/TrkB signaling pathway and inhibition of the RhoA/ROCK2 signaling pathway (Li Y. et al., 2016). In mice models impaired by scopolamine and sodium nitrite, ethanol extracts from the pericarp of *X. sorbifolium* (89.80, 44.90 mg kg^−1^, i g), bunkanka saponins (1.51, 0.76 mg⋅kg^−1^, i g), and ST-n-2 (a saponin, 0.32, 0.16 mg⋅kg^−1^, i g) were found to notably improve memory acquisition after impairment induced by scopolamine and memory consolidation impairment induced by sodium nitrite. The mechanism may involve central cholinergic and glutamatergic nervous system functions and protection against damage caused by reactive oxygen species (ROS) in brain tissue.

In summary, the ability of *X. sorbifolium* to improve learning and memory impairment has been thoroughly studied, and the related active compounds and mechanisms have been revealed. Several studies reported on the signaling pathways that regulate and improve learning and memory impairment, indicating that this is an important pharmacological activity of *X. sorbifolium*. However, this pharmacological activity has not been widely applied in clinical research. Therefore, future research should focus on the practical application of this pharmacological activity to achieve a wide range of clinical applications and maximize the pharmacological value of this plant.

### Anti-Inflammatory Activity

Anti-inflammatories are the second-largest class of drugs after antibacterial agents; thus, the anti-inflammatory effects of the active ingredients of Chinese herbal medicines have become a hot research topic (Li and Zhu, 2012; Rajendiran et al., 2018). The anti-inflammatory effect of *X. sorbifolium* has also been extensively studied, including the anti-inflammatory mechanism behind its traditional uses for rheumatism and scabies.

Current research has found that the extracts and compounds from *X. sorbifolium* mainly affect neuroinflammation, vascular inflammation, and rheumatoid arthritis. The flavonoids and phenylpropanoids isolated from the leaves of *X. sorbifolium* decreased nitric oxide (NO) production in the lipopolysaccharide-induced BV2 microglial cells. Among them, the inhibitory effect of 4-*O*-*β*-D-glucopyranosyl-trans-*p*-coumaric acid (IC_50_ = 9.08 ± 1.23 μM) on NO was significantly stronger than that of the positive control minocycline (IC_50_ = 37.04 ± 2.09 μM) (Li N. et al., 2016). Another report also indicated that a 70% ethanol extract of *X. sorbifolium* husk is rich in effective anti-neuro-inflammatory active ingredients. Among them, the two triterpenoids (IC_50_ values of 5.01 ± 0.22 and 3.05 ± 1.21 μM) and the two alkaloids (IC_50_ values of 9.61 ± 0.21 and 4.72 ± 0.52 μM) were significantly stronger than the positive drug minocycline (IC_50_ = 30.31 ± 3.01 μM) (Chen et al., 2020a). The ethanol extract from *X. sorbifolium* seeds (1–50 μg mL^−1^) has significant implications for the prevention of vascular complications, which is linked to inhibition of the NF-κB/reactive oxygen species (ROS) pathway and activation of the Nrf-2/HO-1 pathway (Jung Joo et al., 2018). Xanthoceraside (extracted from the husk of *X. sorbifolium*) significantly inhibits the release of NO, IL-1β, and TNF-α in a concentration (0.01 and 0.1 µM)-dependent manner (Qi et al., 2013). In addition, gavage with n-butanol extract (2,000 mg kg^−1^; from *X. sorbifolium* wood) has shown a significant inhibitory effect on ear swelling induced by xylene (25 μL·ear^−1^) in Chinese Kunming (KM) mice, indicating that n-butanol extracts in *X. sorbifolium* wood can inhibit the early exudation and edema caused by inflammation (Kuang et al., 2001). Similarly, 7 days of gavage with the n-butanol extracts of *X. sorbifolium* wood (1.5 g kg^−1^) significantly inhibited the swellings in inflamed feet, non-inflamed feet, and forelimbs of Wistar male rats induced by the intradermal injection of Freund’s complete adjuvant (0.1 ml) in the plantar region of the foot. These results indicated that n-butanol extracts from lignum xanthocerais had an inhibitory effect on primary and secondary joint swelling in rats with adjuvant arthritis and improved the systemic symptoms of adjuvant arthritis in rats (Kuang and Liu, 2002).

At present, most reports only used the crude extract to verify the anti-inflammatory activity of *X. sorbifolium*. Although it has been verified *in vivo* and *in vitro*, there are still great shortcomings in the research of *X. sorbifolium*. Therefore, more extensive pharmacological studies should be carried out to clarify the mechanism underlying the anti-inflammatory effect of *X. sorbifolium* and determine its active compounds to provide reliable data to support the development and utilization of *X. sorbifolium*.

### Anti-Tumor Activity

In general, the anti-tumor activities of natural products are often evaluated by their ability to inhibit the proliferation of tumor cells and induce immune cells to secrete cytokines that act on tumor cells (Keawsard et al., 2012). The functional constituents of natural plant resources as anti-cancer agents have become increasingly popular, with many focusing on barrigenol triterpenes.

In one study, xanthoceraside (10 μM) significantly inhibited the proliferation of human melanoma A375. S2 cells through the mitochondrial pathway in a concentration- and time-dependent manner without impairing the viability of normal cells and increased the percentage of cells in the sub-G_1_ phase (Jiao et al., 2014). Moreover, Wang et al. (2016b) adopted the CCK-8 method to test the n-BuOH layer of a 70% *X. sorbifolium* husk extract and 10 barrigenol-like triterpenoids against cancer cells of the human hepatoma cell line (HepG2), human colorectal cancer cell line (HCT-116), and human glioma cell line (U87-MG). The results showed that the n-BuOH layer of a 70% *X. sorbifolium* husk extract exhibited many anti-tumor activities against HepG2, HCT-116, and U87-MG cell lines, with IC_50_ values of 15.3, 6.7, and 16.3 μg mL^−1^, respectively (Wang et al., 2016b). Furthermore, Chan (2007) isolated xanthoceraside from an 80% ethanol extract of *X. sorbifolium* husk, and determined its effect on the growth of various human cancer cell lines, including OVCAR3 (ovary), HTB-9 (bladder), U2OS (bone), DU145 (prostate), K562 (leukocyte), HepG2 (liver), MCF-7 (breast), T98G (brain), HCT116 (colon), H460 (lung), SK-Mel-5 (skin), and HeLa-S3 (cervix) cell lines, using the MTT assay with IC_50_ values of 14.5 ± 1, 48.3 ± 3, 46.7 ± 8, 41.7 ± 8, 44.3 ± 6, 57 ± 11, 65 ± 0, 77.5 ± 11, 103.3 ± 3, 112.5 ± 4, 115 ± 7, and 130 ± 14 μg mL^−1^, respectively.

*Xanthoceras sorbifolium* has an inhibitory effect on a variety of cancer cells. However, CCK-8 and MTT methods can only verify its inhibitory effect but fail to reveal the exact molecular mechanism. Therefore, further *in vivo* experiments are needed to determine the effective chemical constituents and signal pathways and clarify the anti-cancer mechanism of *X. sorbifolium*.

### Antioxidant Activity

Oxidative stress plays a crucial role in the pathogenesis of various chronic diseases, such as diabetes, cardiovascular diseases, and neurodegenerative diseases. ROS are often associated with oxidative stress. Scavenging or inhibiting ROS generation can delay or prevent oxidative cellular oxidizable substrates from achieving anti-oxidation (Uttara et al., 2009; Small et al., 2012; Zhao et al., 2018). In recent years, deeper investigations of *X. sorbifolium* have highlighted its antioxidant activity.

Zhang et al. (2015) reported that the compounds epicatechin, catechin, myricetin, and dihydromyricetin, which exist in lignum xanthocerais, showed remarkable protective effects against peroxyl radical-induced DNA strand scission (when the concentration was 10 μmol L^−1^, the protective rates were 92.10, 94.66, 75.44, and 89.95%, respectively). Furthermore, some researchers have reported that saponins from the *X. sorbifolium* nutshell have a higher scavenging effect than vitamin C *in vitro*. The hydroxyl radical-scavenging effects of saponins were 15.5–68.7% at a concentration of 0.18–2.52 mg mL^−1^ (Zhang and Zhou, 2013a). The antioxidant activity of crude extracts in ethanol extraction fractions of 10, 30, 50, 70, and 95% showed scavenging effects on DPPH with a dose-dependent relationship. The 70% ethanol extract had the most substantial effect (the DPPH scavenging rate reached 70.82% at a mass concentration of 0.2 mg mL^−1^) (Zhang et al., 2017). These results also support the traditional use of treating metabolic syndromes, such as diabetes and hypertension.

As various studies have revealed the antioxidant activities of *X. sorbifolium*, this plant should be further explored for potential novel antioxidants. However, verification methods such as DPPH analysis may overestimate the antioxidant content. Moreover, these determination methods cannot characterize all the analytical properties of the extract (Amorati and Valgimigli, 2015). Therefore, these methods are not yet sufficient for elucidating the antioxidant mechanism of *X. sorbifolium*, and further research is required to investigate the kinetics of this mechanism.

### Other Pharmacological Activities

*Xanthoceras sorbifolium* has other pharmacological activities, including anti-HIV, and plays protective roles in cardiovascular and cerebrovascular diseases (Ma et al., 2000; Li et al., 2007b; Jin et al., 2010; Zhang et al., 2013; Geng et al., 2014). Furthermore, it also inhibits the activities of pancreatic lipase and tyrosinase.

For example, 3-oxotirucalla-7, 24-dien-21-oic acid, oleanolic acid, and epigallocatechin-(4*β→*8,2*β→O*-7)-epicatechin isolated from the methanol extract from lignum xanthocerais are inhibitors of HIV-1 protease with IC_50_ values of 20, 10, and 70 μg mL^−1^, respectively (Ma et al., 2000). Li et al. (2007b) reported that the coumarin compound (cleomiscosin B) extracted from the seed coat possessed strong anti-HIV-1 activity *in vitro*. It also had a strong inhibitory effect on HIV-1 IIIB-induced C8166 cell formation in the syncytia with an EC_50_ value of 8.61–12.76 μg mL^−1^ and a selectivity index of greater than 15.67–23.23 (Li et al., 2007b). Xu et al. (2014) found that xanthoceraside can significantly improve cerebral artery ischemia-reperfusion injury in rats. Its mechanism may promote synaptic remodeling and/or reduce synaptic structural and functional damage (Xu et al., 2014). Geng et al. (2014) found that xanthoceraside significantly inhibits pancreatic lipase activity, and the maximum inhibitory rate can reach 87.5%. Therefore, as a weight-loss factor, xanthoceraside has broad prospects as both healthy food and medicine (Geng et al., 2014). Moreover, flavonoids and saponins extracted from the husk of *X. sorbifolium* have been shown to exhibit inhibitory effects on tyrosinase. For example, the inhibition rate was 45% at a flavonoid concentration of 0.48 mg mL^−1^ and showed non-linear changes (Zhang et al., 2013). Zhang et al. (2013) showed that the saponin extract inhibited tyrosinase and was non-competitive at a concentration of 0.36 mg mL^−1^, where the inhibition rate reached 64.6% (Zhang et al., 2013). Therefore, extracting flavonoids and saponins from the husk of *X. sorbifolium* as whitening components is in line with the current development trend of exploiting natural compounds as beauty components and improves the economic value of agricultural byproducts.

In general, there are many studies on the pharmacological activities of triterpenoids in *X. sorbifolium*. It is worth noting that xanthoceraside has many biological activities, and it may become a candidate compound for the prevention and treatment of AD and related diseases (Chi et al., 2010). The effective parts or active components with anti-AD effects can be isolated from *X. sorbifolium*, which can be used to prepare functional foods or drugs to improve learning and memory and have the potential to become leading anti-AD drugs through further research and development. However, screening for bioactivity and evaluation of most other categories of compounds remains at the crude extract level. To date, only a few reports have investigated the chemical constituents, bioactivity, pharmacodynamic, and mechanisms of action of extracts from *X. sorbifolium*, which remain elusive.

## Structure-Activity Relationships

In the process of summarizing the chemical composition and pharmacology of oleanane-type triterpenes in *X. sorbifolium*, the general structural properties of the extracts along with their biological activities have been investigated. Compounds with the same structural skeletons, but different types or positions of substituents have more significant impacts on cytotoxic activities. The triterpenoids with structural skeletons of R1-barrigenol triterpenes were the active ingredients for anti-AD. Similarly, the barringtogenol C triterpenes and 16-deoxy barringtogenol C triterpenes were the active ingredients for anti-tumor activities. The triterpenes with no hydroxyl substitution at C_15_ and C_16_ showed no activity. Conversely, a hydroxyl substitution at C_15_ and C_16_ or C_28_ by a glycoside group displayed anti-tumor activity *in vitro*. However, if the hydroxyl substitution occurred at C_24_ or glycosylation at C_3_ and C_21_, the anti-tumor activity increased. In addition, angeloyl groups at C_21_ and C_22_ also play a role in inhibiting cell activity (Li 2006a; Wang et al., 2016b).

Regarding the relationship between the anti-AD activity and the structure of the ingredients, the most vigorous activity occurs in the compound R1-barrigenol. This activity disappears when its C_3_ is linked with a sugar group or when C_24_ is substituted with a hydroxyl group. Conversely, if ring A or E is substituted by a hydroxyl group, acetoxy group, or sugar chains, the activity of the compound decreases or disappears because of the different steric hindrance of C_21_/C_22_. For example, in compounds 22-di-*O*-angeloyl-24-hydroxy-R_1_-barrigenol and 21-*O*-angeloyl-24-hydroxy R1-barrigenol, if the angelic acyls substitute with either C_21_ or C_22_, the activity decreases. Therefore, when the activity occurs at C_22_, the activity decreases, and when the activity at C_21_ occurs, the activity vanishes. At both C_21_ and C_22_, the compound exhibits weak activity. Conversely, the substitution of α-hydroxyl at C_15_ or C_16_ can enhance activity (Li, 2006a). A study on the anti-tumor effects showed that saponins with the sugar chains at C_3_ and C_21_ exhibited significant cytotoxicity. When an acetoxy group and C_28_ substituted C_22_ with a hydroxyl group, the activity was enhanced. However, the activity decreased after exchanging the positions in the two substituent groups. Furthermore, the activity does not seem to be affected by C_24_ substitution (Chan, 2007). The structure of barrigenol-like triterpenoids greatly influences their activity. Thus, owing to its unique biological activity, *X. sorbifolium* has significant and far-reaching importance in the development of new natural anti-tumor and anti-AD drugs (Yu et al., 2012a).

## Applications

*Xanthoceras sorbifolium* is a multipurpose plant. All sections of the plant are edible, medicinal, economical, and of ecological value. The trunks and branches, fruits, leaves, and other parts contain natural products with rich structures, including a wide range of biological activities and pharmacological effects. Other sections are utilized as food in China, such as fruit, tea, and cooking oil. *Xanthoceras sorbifolium* has ornamental value and is useful for carbon storage, soil remediation, and water conservation. The plant can also be used as industrial raw materials.

### Traditional Applications

As a traditional medicinal herb in China, *X. sorbifolium* has been used widely in traditional Chinese and Mongolian medicines. Various plant parts are used for medicine, including trunks and branches, leaves, fruits, seeds, and flowers. The different parts have different medicinal values. The trunks and branches, lignum xanthocerais, are also called “xi la sen deng” in traditional Mongolian medicine (Pharmacopoeia Committee of the Ministry of Health of the People’s Republic of China, 1998). The therapeutic significance of lignum xanthocerais has been well acknowledged in the ancient Mongolian classics such as “*Jing Zhu Ben Cao*” (Qing Dynasty, AD 1848), “*Meng Yao Zheng Dian*” (Qing Dynasty, AD 19th), and “*Chinese Materia Medica*.” (Demar, 1986) In the 1977 edition of the “Chinese Pharmacopeia,” the folk remedies for the treatment of rheumatism with *X. sorbifolium* leaves were first recorded (Commission NP. 1977). It has been reported that it is sweet-flavored cool-natured, and suitable to treat scurvy, rheumatism, rheumatoid arthritis, enuresis in children, rheumatic heart disease, swollen glands, overheating, and swelling, and also offers pain relief (Chinese Materia Medica Editorial Committee, 2004; Wang et al., 2011). In addition, fruits are used to treat rheumatism, gout, and enuresis in children as a folklore medicine in Inner Mongolia. It has been developed into a product named “Pediatric Urinary Suspension” by pharmaceutical companies (Zhao et al., 2008). According to the *new Tibetan medicine formula*, lignum xanthocerais is used in Liuweiximi pills in Tibet, China. It has the effects of tonifying the kidney, “expelling wind and dampness,” relieving pain, and treating kidney and low back pain as well as frequent urination caused by kidney cold (Institute of Tibetan medicine, 1975). *X. sorbifolium* is also widely used in Northeast China. For example, its seeds are used to treat nocturia in children (Xie, 1996). Its fruit is mainly used for rheumatoid arthritis (Ministry of Health of Shenyang Army Logistics Department, 1970); its wood can dispel wind, remove dampness, detumescence, and relieve pain (National Administration of Traditional Chinese Medicine, 1999).

In summary, *X. sorbifolium* has a wide range of traditional uses, and most of its recorded traditional applications are concentrated in northern China. Effectively combining traditional applications of *X. sorbifolium* with modern clinical applications will be a notable future research direction.

### Clinical Applications

*Xanthoceras sorbifolium* is rich in 278 compounds, providing a reasonable basis for medicinal use. The triterpenes isolated from the husk are promising candidates for medicines to prevent or cure human cancer, AD, enuresis, urinary incontinence, dementia, and modulate cerebral functions (Ge and Wu, 1997; Liu et al., 2007a; Liu et al., 2007b; Chi et al., 2009; Lu et al., 2012; Li and Sun, 2019). The leaves are rich in saponin, flavonoids, protein, and trace elements, with a high inhibitory effect on various human tumor cells (such as breast cancer, prostate cancer, gastric cancer, liver cancer, cervical cancer, and leukemia). The leaves can improve the functions of the central nervous system, cholinergic nerve system and fight the damage caused by free radicals, assisting the treatment of urine incontinence and an overactive bladder (Si, 1996). After degreasing, the kernels of *X. sorbifolium* can be made into efficient drugs to treat pediatric enuresis. The data from 100 initial clinical results show that its efficacy rate is as high as 93%. In addition, lignum xanthocerais is often combined with other medicines in clinical preparations to treat skin diseases and rheumatism. The traditional and modern prescriptions of lignum xanthocerais are listed in Table 3.

**TABLE 3:** Traditional and modern prescriptions of lignum xanthocerais in China.

NO.	Parts used	Preparation name	Mode of preparation	Traditionaland clinical uses	References
1	Wood	Sendeng Siwei Tangsan	Decoction	Arthritis and edema	Commission NP (1977); Li et al. (2013b)
2	Wood	WenGuanMu Ershiwuwei Wan	Pill	Relieve rheumatic pains, remove paralysis, anti-inflammation	Ba (2007)
3	Wood	WenGuanMu Jiuwei Decoction	Decoction	Relieve rheumatic pains, detumescence and purging fire	Ba (2007)
4	Wood	WenGuanMu Sanwei Decoction	Decoction	Relieve rheumatic pains, clear heat and detoxify, anti-inflammation, moisturize skin	Si (1996)
5	Wood	WenGuanMu Ruangao	Uunguent	Used for psoriasis, neurodermatitis, and other skin diseases	Commission NP (1977)
6	Wood	Sendeng·Ji ri gan Decoction	Decoction	Used for rheumatoid arthritis and brucellosis	Meng (1991)
7	Wood	Sendeng·Manmari	Pill	Relieve rheumatic pains, clear heat and detoxify. Used for rheumatism and scabies	Meng (1991)
8	Wood	Sendeng·nai ma	Pill	Relieve rheumatic pains, clear heat and detoxify. Used for rheumatism, brucellosis, and scabies	Meng (1991)
9	Wood	Sendeng·Duriben Decoction	Decoction	Relieve rheumatic pains, clear heat and detoxify. Used for rheumatic fever	Meng (1991)
10	Wood	Shendeng Handa	Unguent	Clear heat. Used for Rheumatism, rheumatoid, joint swelling and pain, eczema and other skin diseases	Inner Mongolia Autonomous Region Health Department (1984)

Except for the anti-inflammatory activity, which has been widely used in clinical applications, most of the other pharmacological activities have only been studied theoretically; thus, they lack extensive practical research. Therefore, applying the pharmacological activity of *X. sorbifolium* to clinical practice should be the focus of future *X. sorbifolium* research.

### Edible Applications

The food value of *X. sorbifolium* is mainly derived from its seeds, kernels, and leaves. Edible oil can be extracted from the seeds and the oil ratio is 30.4% in the seeds and 55–66% in the kernel (Yan, 2007). The oil is a cooking oil with a high smoke point, a yellowish color and delicious flavor and may help in preventing cardiovascular and cerebrovascular diseases. In the seed oil, the unsaturated fat has been isolated, accounting for 94.0%, including linoleic acid (36.9%) and oleic acid (57.16%). (Yan, 2007; Zeng et al., 2013). The tender kernels have a unique fruit flavor that can be eaten raw or processed into canned food for giving to infants during weaning.

Additionally, kernels can also be processed into a nutritious fruit juice and a high-quality protein drink. Moreover, the seeds are delicious when fried (Li et al., 2003). The leaves of *X. sorbifolium* can be processed for tea and lower blood lipids, blood pressure, and protect the cardiovascular and cerebrovascular vessels. In tea, the protein content is as high as 19.8–23.0%, which is higher than black tea, and the caffeine content is similar to scented tea (Hua, 2004). The flower is a hardy honey plant with a long flowering period, enabling a large amount of honey to be produced from the flowers. The husk remaining after oil extraction can be made into high-protein beverages (Li et al., 2003).

### Other Applications

In addition to its edible and medicinal uses, *X. sorbifolium* has a high economic and ecological value. The plant is a potential bio-energy feedstock plant and has been identified as a major woody energy species for biodiesel production. Producers receive special support from the Chinese government for its development. The whole plant can be used as an eco-friendly tree species for soil and water conservation and land reclamation in mining areas (Bai et al., 2010). The husk contains 12.2% furfural, which is the best raw material for furfural extraction. Husks and seed coats can be used as a source of activated carbon, xylitol, alcohol, and other chemical raw materials. The trunks and branches can be exploited as top-grade furniture and farm tools because of the hard texture, strong corrosion resistance, and dark maroon color with a beautiful vein pattern (Xu and Yu, 2010). The plant has a long flowering period with bright colored flowers making it highly ornamental. As an ornamental tree, it is suitable for planting in gardens, parks, and scenic areas (Wan et al., 2010). The flowers are also edible, and the pollen and oil can be used to make advanced beauty skincare products (Zhang et al., 2012).

## Conclusion and Discussion

In conclusion, *X. sorbifolium*, a native plant with economic and medicinal value in China, is rich in resources and is widespread throughout northern China. Here, *X. sorbifolium* was reviewed with regard to botany, phytochemistry, pharmacological activity, structure-activity relationship, and applications. Concerning the phytochemistry of *X. sorbifolium*, a total of 278 compounds have been discovered: 124 terpenoids, 48 flavonoids, 14 phenylpropanoids, 17 steroids, 17 phenols, 29 fatty acids, 9 alkaloids, 4 quinones, and 16 other compounds. Modern pharmacology has gradually verified the traditional efficacy of *X. sorbifolium* and explored its role in treating AD, rheumatism, vasculitis, scabies, and other diseases. The pharmacological effects have mostly focused on improving learning and memory impairment, as well as on anti-inflammatory and antioxidant effects. Nevertheless, there are still some research barriers that need to be overcome. Despite numerous studies on the chemical constituents of *X. sorbifolium*, research into the corresponding pharmacological activities predominantly involves terpenoids and saponins, especially the landmark compound xanthoceraside, which shows good pharmacological activity related to improving learning and memory impairment, anti-inflammation, and anti-tumor properties. However, research into other types of compounds is very limited. Moreover, it is difficult to link the phytochemistry and pharmacological effects of *X. sorbifolium*; therefore, this should mark the main direction of future *X. sorbifolium* research. Furthermore, in the process of studying the structure-activity relationships of *X. sorbifolium*, it was discovered that the biological activities of compounds with the same structural skeleton but different substituent positions have significant differences, especially in their anti-tumor and anti-AD properties. More research into the structure-activity relationships of *X. sorbifolium* will be highly significant for the development and utilization of these compounds. In addition, no studies have yet reported any differences in the main production areas or seasons for the same plant component.

Despite the substantial practical value of *X. sorbifolium*, current research is not comprehensive. It requires further analysis of five main aspects to fully understand all the characteristics of *X. sorbifolium*. First, 278 compounds were isolated from *X. sorbifolium*, most of which were terpenoids. However, a lot of unknown compounds are yet to be found. The bioactivity-oriented separation strategy can be used to study potential phytochemicals and explore target compounds. However, the difficulty of phytochemical separation and the low content of compounds limit drug development. In general, using abundant phytochemicals to develop potential compounds can lay a foundation for developing new drugs. Some active derivatives should be considered to enrich the medicinal value of *X. sorbifolium*. Second, there is currently no standard quality control method or index for assessing the components of *X. sorbifolium*. Therefore, considerable research should be devoted to creating a standard quality assessment approach to ensure the quality of *X. sorbifolium.* Specifically, it is necessary to determine *X. sorbifolium* contents or produce standardized fingerprints to index the components of this species. Third, research on the biological activity of the compounds remains limited, with the majority of selected biological activity research employing only the crude extract. Therefore, more studies are required to assess the pharmacodynamic material and pharmacological mechanisms to obtain relevant compounds responsible for the pharmacological effects and unveil the potential mechanisms involved. In addition, research on the antioxidant activity of *X. sorbifolium* is mostly based on chemical methods such as DPPH experiments, which are not particularly thorough, making it difficult to reveal the antioxidant mechanism of *X. sorbifolium*. We believe that *X. sorbifolium* can become an excellent antioxidant; however, sufficient *in vivo* and *in vitro* studies are required to support this development. Fourth, additional pharmacokinetic, metabolomic, and clinical studies are required to elucidate all chemical constituents entering the body and their processes within the body. Such research would aim to build a bridge between the chemical constituents and the systemic clinical effects, which is crucial for fully understanding the target components, pharmacological effects, and potential applications of this plant. Fifth, at present, there is little comprehensive utilization of *X. sorbifolium* resources, especially the research and utilization of tea-making technology, drinking methods, and health products based on the leaves, which still have a great potential for development and value-added utilization. Furthermore, the oil production from *X. sorbifolium* generates large volumes of waste, including husks, oil residue, seed meals, and seed coats. Research has shown that the residues are rich in various compounds. There is an urgent need to create new technological systems to develop and utilize these waste products to add value and create societal benefits.

In summary, this review provides a comprehensive and critical analysis of the phytochemistry, pharmacology, and traditional and modern applications of *X. sorbifolium*. We also discuss the limitations of existing literature and propose solutions for further research and development. Finally, we summarize and analyze the importance of *X. sorbifolium* for medicinal applications.

## Data Availability

The original contributions presented in the study are included in the article/Supplementary Material, further inquiries can be directed to the corresponding author.
